# ECM1 and ANXA1 in urinary extracellular vesicles serve as biomarkers for breast cancer

**DOI:** 10.3389/fonc.2024.1408492

**Published:** 2024-07-08

**Authors:** Hai Huang, Jingyu Wan, Xudong Ao, Shuang Qu, Meng Jia, Keyu Zhao, Junqing Liang, Ke Zen, Hongwei Liang

**Affiliations:** ^1^ State Key Laboratory of Pharmaceutical Biotechnology, School of Life Science, Nanjing University, Nanjing, China; ^2^ Department of Emergency, Nanjing Drum Tower Hospital, School of Life Science and Technology, China Pharmaceutical University, Nanjing, China; ^3^ Peking University Cancer Hospital, Affiliated Cancer Hospital of Inner Mongolia Medical University, Hohhot, China

**Keywords:** ECM1, ANXA1, urinary extracellular vesicles, biomarker, breast cancer

## Abstract

**Objective:**

Although urinary extracellular vesicles (uEVs) have been extensively studied in various cancers, their involvement in breast cancer (BC) remains largely unexplored. The non-invasive nature of urine as a biofluid and its abundant protein content offer considerable potential for the early detection of breast cancer.

**Methods:**

This study analyzed the proteomic profiles of uEVs from BC patients and healthy controls (HC). The dysregulation of ECM1 and ANXA1 in the uEVs was validated in a larger cohort of 128 BC patients, 25 HC and 25 benign breast nodules (BBN) by chemiluminescence assay (CLIA). The expression levels of ECM1 and ANXA1 were also confirmed in the uEVs of MMTV-PyMT transgenic breast cancer mouse models.

**Results:**

LC-MS/MS analysis identified 571 dysregulated proteins in the uEVs of BC patients. ECM1 and ANXA1 were selected for validation in 128 BC patients, 25 HC and 25 BBN using CLIA, as their fold change showed a significant difference of more than 10 with *p*-value<0.05. Protein levels of ECM1 and ANXA1 in uEVs were significantly increased in BC patients. In addition, the protein levels of ECM1 and ANXA1 in the uEVs of MMTV-PyMT transgenic mice were observed to increase progressively with the progression of breast cancer.

**Conclusion:**

We developed a simple and purification-free assay platform to isolate uEVs and quantitatively detect ECM1 and ANXA1 in uEVs by WGA-coupled magnetic beads and CLIA. Our results suggest that ECM1 and ANXA1 in uEVs could potentially serve as diagnostic biomarkers for breast cancer.

## Introduction

Breast cancer is a significant global health burden, with two million cases diagnosed and 15.5% of deaths attributable to breast cancer (1). The high mortality rate of breast cancer can be attributed to several factors, including modern lifestyle factors, environmental conditions, delayed diagnosis, lack of sensitive and specific markers and lack of awareness in the general population (2). Triple negative breast cancer (TNBC), which accounts for 15–20% of incident breast cancers, is the only breast cancer subtype that lacks targeted treatments (2, 3). Using clinical assays, TNBC is human epidermal growth factor receptor 2 (HER2) negative and has <1% expression of estrogen receptors (ER) and progesterone receptors (PR) by immunostaining (2). Patients with TNBC do not respond to endocrine therapy or HER2-targeted therapy, and treatment is based on a combination of commonly used breast cancer therapies, including surgery, radiation and chemotherapy (2, 3). Lymphovascular invasion (LVI) is an early event in the development of metastasis and is a potent prognostic factor (3, 4). The presence of LVI also affects the selection of adjuvant therapy, such as radiotherapy and chemotherapy (3, 4). Therefore, early detection of breast cancer and identifying biomarkers that can predict TNBC and LVI can significantly reduce mortality through optimal medical treatment and intervention (2). However, conventional diagnostic methods like mammography, ultrasound and magnetic resonance imaging have limitations in terms of accessibility, invasiveness and accuracy (2). Mammography, which is considered the gold standard for early detection of breast cancer, is associated with risks such as overdiagnosis, overtreatment, false-positive results, radiation exposure and unnecessary biopsies (2, 5). Immunohistochemistry is a common technique used in breast biopsies, whether surgical or needle-based, to evaluate the LVI and the expression of ER, PR and HER2 (2, 5). Immunohistochemistry plays a crucial role in the diagnosis of BC, molecular subtyping and the development of personalized treatment strategies. However, it has several drawbacks, including invasiveness, unnecessary testing for benign nodes, potential complications, tumor displacement, risk of metastasis, subjective interpretation leading to result variability, and time-consuming procedures that may delay timely treatment initiation (2, 3). Therefore, discovering non-invasive and reliable diagnostic biomarkers for breast cancer is urgently needed.

Extracellular Vesicles (EVs) are released by different cell types and play a crucial role in intercellular communication (6). They transfer signaling components, such as proteins, nucleic acids, lipids and transcription factors, thereby influencing various biological processes in recipient cells (7, 8). The EVs originating from cancer cells may contain molecules linked to cancer progression and invasion, promoting intercellular cargo transfer within the tumor microenvironment (7, 9). Furthermore, the EV cargo content may reflect the altered state of the original tumors, and their concentrations have been correlated with cancer progression (8). EVs have been found in different body fluids, such as blood and urine, and have the potential to be used as diagnostic biomarkers for diseases, especially tumors (10). Although it is possible to assess circulating EVs through blood biopsy, it is still an invasive procedure. Urine is a non-invasive biofluid that can be used for clinical diagnosis. Research has shown that it can serve as a valuable resource for biomarkers in several diseases, including kidney, bladder, urinary tract infections and numerous oncological conditions (11). uEVs have been identified as potential non-invasive biomarkers for several urological and non-urological cancers, Parkinson’s disease and Alzheimer’s disease (12, 13). Recent studies have identified differentially expressed proteins and RNAs in the uEVs of breast cancer patients (14–18). In our previous study, we introduced an innovative, user-friendly and highly efficient technique for the capture and quantitative analysis of EVs by leveraging wheat germ agglutinin (WGA)-coupled magnetic beads in conjunction with flow cytometry (19).

In this study, we developed a simple and efficient method for isolating and quantitatively detecting specific proteins in uEVs from breast cancer patients. The method employs WGA coupled with magnetic beads and CLIA. The results of the study indicate that uEVs, which contain ECM1 and ANXA1, have the potential to be used as diagnostic biomarkers for breast cancer.

## Materials and methods

### Clinical samples

**Figure 1:**
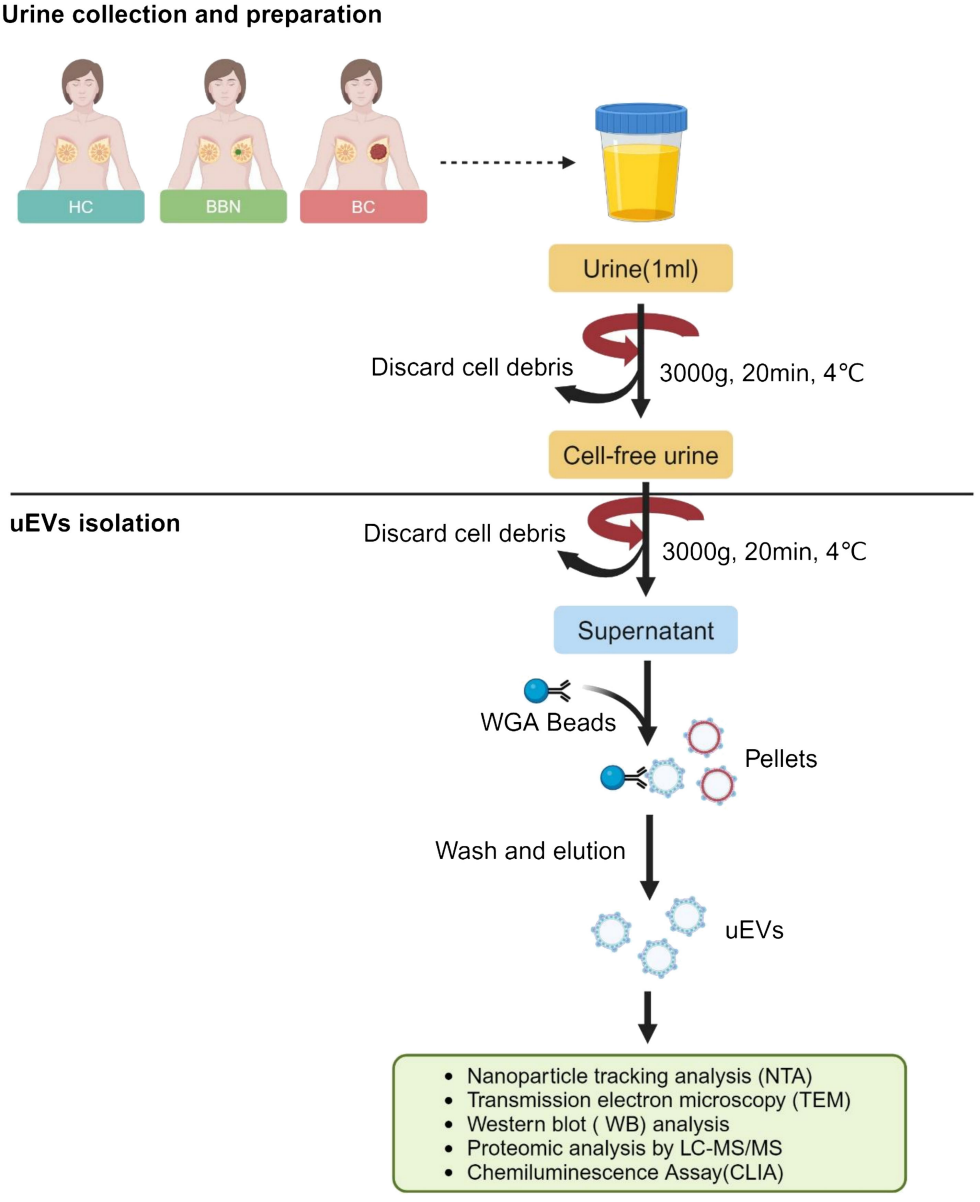
Workflow for the investigation of the uEV proteome. (Created with BioRender.com).

The objective of this pilot study was to identify protein biomarkers in uEVs for the diagnosis of breast cancer. The study procedure is illustrated in **Figure 1**. A total of 50 mL midstream urine samples were randomly collected and placed in sterile containers. Samples were collected from 128 newly diagnosed BC patients before surgery at Peking University Cancer Hospital (Inner Mongolia Campus) and the Affiliated Cancer Hospital of Inner Mongolia Medical University. Urine samples were collected from healthy women without a history of cancer who were age-matched to the BC patients (n = 25). Additionally, samples were collected from women with benign breast nodules prior to IHC of breast biopsies by either surgical or needle procedures, also age-matched to the BC patients (n = 25). **Table 1** displays the clinical characteristics of HCs, BBNs and BCs. All participants provided written informed consent, and the study protocol was reviewed and approved by the Ethics Committee of Peking University Cancer Hospital (Inner Mongolia Campus) and the Affiliated Cancer Hospital of Inner Mongolia Medical University (approval number: KY202315).

**Table 1:** Clinical characteristics of healthy controls, benign breast nodules and breast cancer patients.

		healthy volunteers	benign breast nodules	breast cancer
**n**		25	25	128
**Age**		56.55 (18.75)	54.65 (19.06)	52.58 (14.29)
TNM				
T				
	**1**			57
	**2**			71
N				
	**0**			57
	**1**			71
M				
	**0**			128
Lymphovascular invasion				
	**Negative**			73
	**Positive**			55
triple-negative breast cancer				
	**YES**			28
	**NO**			100

### Animal

FVB/NJGpt mice (strain No. N000026) and FVB/NJGpt-Tg (MMTV-PyMT)/Gpt mice (strain No. T004993) were obtained from GemPharmatech (Nanjing, China) at five weeks of age. The male MMTV-PyMT mice (FVB/NJGpt-Tg (MMTV-PyMT)/Gpt mice) were randomly bred with homozygous FVB females to obtain F1 female mice (hereafter MMTV-PyMT mice) heterozygous for the MMTV-PyMT transgene. These mice developed breast cancer and were used as cases. FVB homozygous females were used as controls. The MMTV-PyMT mice were selected for the study of the changes in the breast cancer diagnostic markers ECM1 and ANXA1 in uEVs during the development of breast cancer. Prior to the commencement of the experiments, all mice were quarantined for one week and tested negative for specific pathogens. Urine samples were collected from MMTV-PyMT and FVB mice at three weeks of age and continued weekly. All animal handling and care procedures were conducted in accordance with the National Institutes of Health Guide for the Care and Use of Laboratory Animals and were approved by the Ethical Committee of Nanjing University (Nanjing, China). The mice were maintained in a controlled environment with a humidity of 50 ± 10%, a 12/12 h light/dark cycle, and a temperature of 23 ± 2°C. They were provided with unrestricted access to standard chow (catalog number: 1010007, Xietong Bio, Nanjing, China) and water ad libitum.

### Urine preparation

Once the 50 mL of urine samples had been collected from healthy controls, benign breast nodules (BBN) patients and breast cancer (BC) patients, the samples were centrifuged at 3,000 g for 20 minutes at 4°C in order to remove cells, cell debris and bacteria. The resulting supernatants were then collected and stored at -80°C for subsequent analysis.

To collect the urine of the mice, the animals were placed into metabolic cages (Tecniplast Metabolics, Buguggiate, Italy) without access to food until 1 mL of the urine samples were collected. During urine collection, the mice had libitum access to water. The urine samples were centrifuged at 3,000 g for 20 minutes at 4°C once they were collected. Subsequently, the supernatants were collected and stored at -80°C until further analysis.

### Isolation of uEVs by WGA-coupled magnetic beads

The urine samples were subjected to centrifugation at 3,000 g for 20 minutes prior to isolation. Once the WGA-coupled magnetic beads (C995Y30, Thomas Scientific) had been separated, they were incubated with 1 mL urine samples in appropriate tubes at room temperature for 1 h. Subsequently, the tube containing the bead-sample mixture was placed on a magnet and left for 5 minutes to allow the EVs bound to the beads to separate from the supernatant, which was discarded. Subsequently, the tube was removed from the magnet and the EVs bound to the beads were washed twice with phosphate-buffered saline (PBS).

### Characterize the uEVs isolated by WGA-coupled magnetic beads by transmission electron microscopy and nanoparticle tracking analysis

For TEM and NTA, the uEVs were eluted from the WGA-coupled magnetic beads using an elution buffer containing 500 mM N-acetyl-D-glucosamine. The concentration and size distribution of the eluted uEVs were measured using a transmission electron microscope (JEOL JEM 2010) and a NanoSight NS300 (Malvern Panalytical Ltd, Malvern, UK).

For the TEM assay, the uEV pellets were fixed with 3 μL of 2.5% glutaraldehyde for 30 minutes at room temperature. Subsequently, 3 μL of each sample was applied to carbon/formvar-coated grids and incubated for 10 minutes. The grids were then washed twice with PBS for 3 minutes and 10 times with distilled water for 2 minutes. Each uEV sample was negatively stained with 2.5% uranyl acetate for 10 minutes and then allowed to dry overnight at room temperature. The transmission electron microscopy (JEOL JEM 2010) was employed to visualize the uEVs.

For the NTA assay, each sample was diluted in sterile water in two independent dilutions at a recommended concentration of 10^7^-10^9^ particles/mL, or approximately 20-100 particles/frame. The mixture was then infused into the Nanoparticle Tracking Analyzer using a syringe pump set at 50 rpm. The experiment involved the recording of each replicate in five 30-second videos at a controlled temperature of 25°C, a camera level of 14, and a detection threshold of 6. The data was then analyzed using the NanoSight NTA 3.0 software.

### Western blotting analysis WGA-coupled magnetic beads isolated uEVs

The WGA-coupled magnetic bead-bound uEVs (3×10^8^ particles, amounting to 30 μg protein) were lysed using Radioimmunoprecipitation assay buffer (RIPA buffer) (R0278, Merck). Subsequently, the samples were combined with a 1/5 volume of 5×loading buffer (P0015, Beyotime), heated to 100°C for 5 minutes, and then maintained at -20°C for 5 minutes. Finally, the mixtures of uEV proteins and WGA-coupled magnetic beads were subjected to centrifugation at 10,000 g for 10 minutes. Subsequently, the supernatant was removed, and the samples were separated using the TGX Stain-Free FastCast Acrylamide Kit, 12% (161-0185; Bio-Rad) with running conditions of 80 V in the stacking gel for 20 minutes and 120 V in the resolving gel for one hour. Subsequently, the proteins were transferred to polyvinylidene difluoride membranes and blocked with 5% non-fat milk in Tris-buffered saline containing 0.1% Tween 20 (TBS-T) for one hour at room temperature. Following three washes with TBS-T for 10 minutes each, the membranes were incubated overnight at 4°C with primary antibodies (diluted 1:1000). The following primary antibodies were used: CD9 (20597-1-AP, Proteintech), TSG101 (28283-1-AP, Proteintech), CD63 (52090S, CST), ALIX (12422-1-AP, Proteintech), GM130 (11308-1-AP, Proteintech), ECM1 (11521-1-AP, Proteintech), and ANXA1 (21990-1-AP, Proteintech). Subsequently, the membranes were washed three times with TBS-T for 10 minutes and then incubated for 2 hours at room temperature with horseradish peroxidase-conjugated secondary antibodies (ab205718, abcam) (diluted 1:2000). The protein bands were then detected using the SuperSignal™ West Dura Extended Duration Substrate (34075, Thermo Fisher Scientific) after another round of washing. Subsequently, the samples were imaged using a chemiluminescence imager (Alliance Q9 Advanced, UVITEC).

### Proteomic analysis of the uEVs isolated by WGA-coupled magnetic beads

The uEVs were bound to magnetic beads and coupled with WGA, and the resulting protein samples were lysed in accordance with the instructions provided by the EasyPep Maxi MS Sample Prep Kit (A45734, Thermo Scientific). The samples were then digested with sequencing grade porcine trypsin (1:20 ratio) for 16 hours at 37 °C. Subsequently, the tryptic peptides were dried using a high-speed vacuum concentrator and resuspended in 0.1% formic acid for LC-MS/MS analysis. The tryptic peptide samples were analyzed using an UltiMate 3000 nano/capillary LC system (Thermo Scientific) coupled to an Impact II™ hybrid quadrupole time-of-flight mass spectrometer (Bruker Daltonics) equipped with a nano-captive spray ion source. The peptide digests were enriched on a μ-precolumn (C18 PepMap 100; Thermo Scientific) and separated on an Acclaim PepMap RSLC analytical column (C18, nanoViper; Thermo Scientific). The C18 column was placed in a column oven that was thermostatically controlled at 60°C. A gradient of 5% to 55% solvent B (0.1% formic acid in 80% acetonitrile) was employed to elute the peptides at a constant flow rate of 0.30 μL/min for 30 minutes. Electrospray ionization was performed at 1.6 kV using the CaptiveSpray system (Bruker Daltonics). Nitrogen was employed as the drying gas, with a flow rate of approximately 50 L/h. Collision-induced dissociation product ion mass spectra were acquired utilizing nitrogen as the collision gas. Mass spectra (MS) and MS/MS spectra were obtained in the positive ion mode at a frequency of 2 Hz over a range (m/z) of 50-2200. The collision energy was adjusted in accordance with the m/z value, with each sample being run in triplicate. Cysteine residues were carbamidomethylated as a fixed modification, while methionine oxidation and protein N-terminus acetylation were variable modifications. Protein identification required peptides with at least seven amino acids and at least one unique peptide. The false discovery rate (FDR) of the identified proteins was set at 1% and estimated using reverse search sequences. Additionally, the maximum number of modifications per peptide was limited to five.

For the purposes of proteomic analysis, functional gene ontology and pathway enrichment analysis was performed using the online tool Enrichr and STRING. This tool employs lists of differentially expressed proteins to predict biological processes and pathways. The statistical significance of these predictions was determined using Fisher’s exact test. Additionally, upstream regulation and protein networks were evaluated using Ingenuity Pathway Analysis, based on the differentially expressed proteins identified in the plasma experiment. All statistical tests were conducted in a two-tailed manner, and a p-value of less than 0.05 was considered to be statistically significant.

### CLIA of the uEVs isolated by WGA-coupled magnetic beads

To prevent nonspecific binding, the bead-bound uEVs were treated with phosphate-buffered saline (PBS) containing 10% bovine serum albumin (BSA) for 10 minutes. Subsequently, primary antibodies targeting ECM1 (11521-1-AP, Proteintech) and ANXA1 (21990-1-AP, Proteintech) were incubated with the bead-bound uEVs for 1 hour at room temperature. Subsequently, the tube was returned to the magnet and the supernatant was discarded. The bead-bound uEVs were then washed twice with PBS (containing 10% BSA) in order to remove any unbound antibodies. Acridinium-ester (CS26015 AAT, Bioquest) labelled goat anti-rabbit IgG (H+L) secondary antibody (31210, Invitrogen) was added and incubated for one hour at room temperature. The bead-bound uEVs were then washed twice with PBS before performing CLIA using a UniCel DxI 800 (Beckman Coulter Life Sciences, USA).

### Statistical analysis

The data were analyzed using GraphPad Prism 8.0. Statistical analyses were conducted using Student’s t-tests and one-way ANOVAs with Tukey’s test, with consideration given to the sample distribution and variance. Results were considered significant at p values of less than 0.05.

## Results

### Characterization of uEVs isolated by WGA-coupled magnetic beads

**Figure 2:**
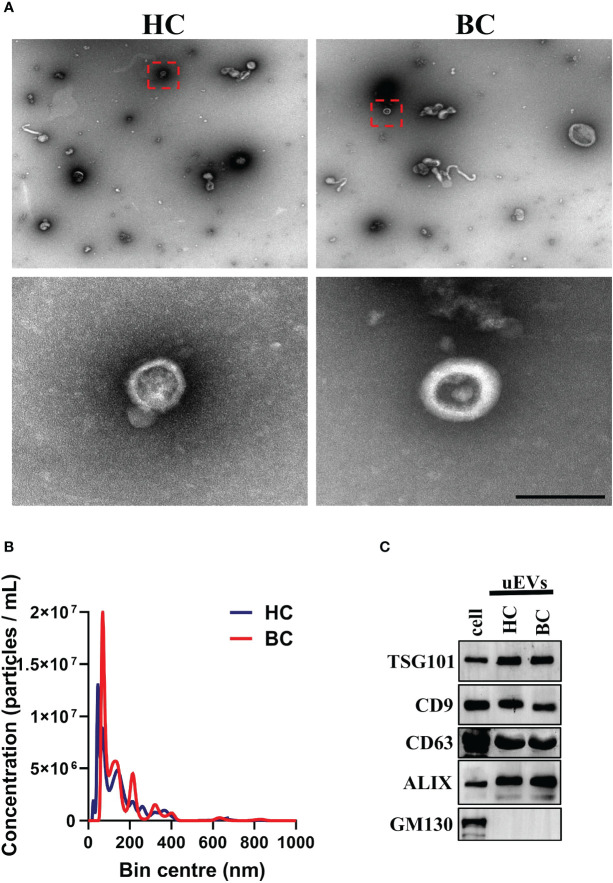
Isolation and identification of uEVs. **(A)** TEM analysis of uEVs isolated by WGA-coupled magnetic beads. Scale bar 100 nm. **(B)** NTA quantification of uEVs isolated by WGA-coupled magnetic beads. **(C)** Western blotting analysis of uEVs (30 μg) isolated by WGA-coupled magnetic beads and cell lysate (60 μg), uEV markers CD63 were used as loading controls. Data from three independent experiments.

Previous research has demonstrated that EVs are rich in glycoproteins (20–24). Lectins, which are carbohydrate-binding proteins that recognize specific glycans, can efficiently label EVs through their major carbohydrate-recognition domains. WGA is an example of such lectins (25). We previous introduced an innovative, user-friendly and highly efficient technique for the capture and quantitative analysis of EVs by leveraging WGA-coupled magnetic beads in conjunction with flow cytometry (19). In order to further simplify the operation process and cost, we developed a novel platform for uEVs capture and detection by WGA-coupled magnetic beads and CLIA (**Supplementary Figure S1**). The uEVs were isolated using WGA-coupled magnetic beads and eluted with an elution buffer containing 500 mM N-acetyl-D-glucosamine. The isolated uEVs were then characterized by TEM, NTA and Western blotting. These results are consistent with previous studies (26), the uEVs displayed a bilayer membrane and an average diameter of 100 nm (**Figures 2A, B**; **Supplementary Figure S2**). The isolated uEVs also contained several uEV marker proteins, including CD9, TSG101, ALIX and CD63, while lacking the marker for membrane-bound vesicles from the Golgi apparatus, GM130 (**Figure 2C**). In conclusion, a simple and effective technique for capturing uEVs using WGA-coupled magnetic beads has been successfully developed, allowing for rapid and reliable analysis.

### The comparison among the uEVs proteomes

**Figure 3:**
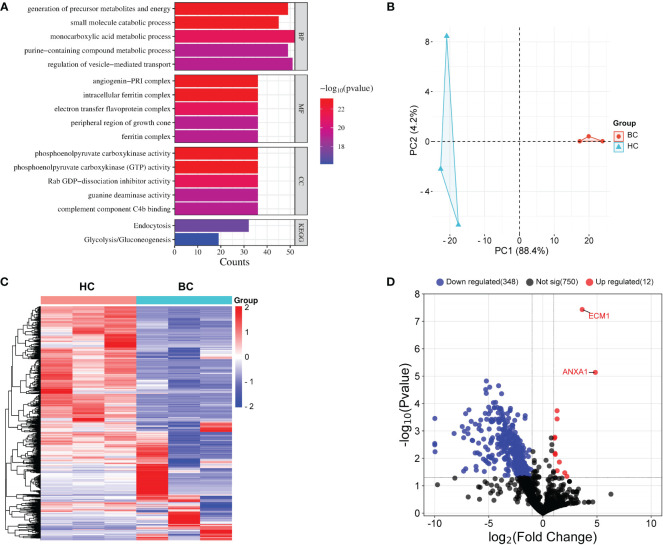
Proteomic analysis of uEVs from BC patients and HC. **(A)** PANTHER analysis performed a Gene Ontology (GO) and KEGG annotation of the identified DEPs. **(B)** PCA analysis. **(C)** Heat map of uEVs differential proteins. Blue represents decreased proteins; red represents increased protein. Tumor, patients with breast cancer; HC, healthy controls. **(D)** Volcanic plot analysis. uEVs protein are significantly different between breast cancer patients and healthy control. Blue represents proteins that are decreased in uEVs of breast cancer patients compared with healthy control; Red represents proteins that are increased in uEVs of breast cancer patients compared with healthy control; Black indicates proteins that did not show changes in uEVs in breast cancer patients compared with healthy control.

A total of 20 breast cancer patients and age-matched HCs were randomly selected and their uEVs were isolated using WGA-coupled magnetic beads. Proteomic analysis using LC-MS/MS of total uEV proteins revealed 571 dysregulated proteins (12 upregulated and 550 downregulated) (**Supplementary Table S1**). The PANTHER database was used to identify the Gene Ontology (GO) analysis and Kyoto Encyclopedia of Genes and Genomes (KEGG) pathways of the dysregulated proteins. The proteins that were dysregulated are primarily involved in small molecule breakdown, precursor metabolite and energy generation, monocarboxylic acid metabolism, regulation of vesicle-mediated transport and purine-containing compound metabolism (**Figure 3A**). The KEGG pathway analysis indicates that these dysregulated proteins are associated with endocytosis and glycolysis/gluconeogenesis (**Figure 3A**). The network analysis was also performed by STRING. Four nodes were identified for the dysregulated proteins (**Supplementary Figure S3**). Principal component analysis (PCA) demonstrated that proteins in uEVs isolated by WGA-coupled magnetic beads could differentiate between BC patients and HCs (**Figure 3B**). A heatmap was generated using the stratified clustering of proteins in uEVs as the horizontal coordinate and the stratified clustering of BCs and HCs as the vertical coordinate, which demonstrated a consistent trend of differentially expressed proteins within the same group (**Figure 3C**). A volcano plot was created using log_2_FC and P-value as the horizontal and vertical coordinates to visualize the results (**Figure 3D**). Two proteins, ECM1 and ANXA1, were identified and subsequently further validated in a larger cohort by CLIA since the fold change of ECM1 and ANXA1 were greater than 10 and a p-value less than 0.001 (**Supplementary Table S1**; **Figure 3D**).

### The protein level of ECM1 and ANXA1 in the uEVs serve as breast cancer diagnostic markers

**Figure 4:**
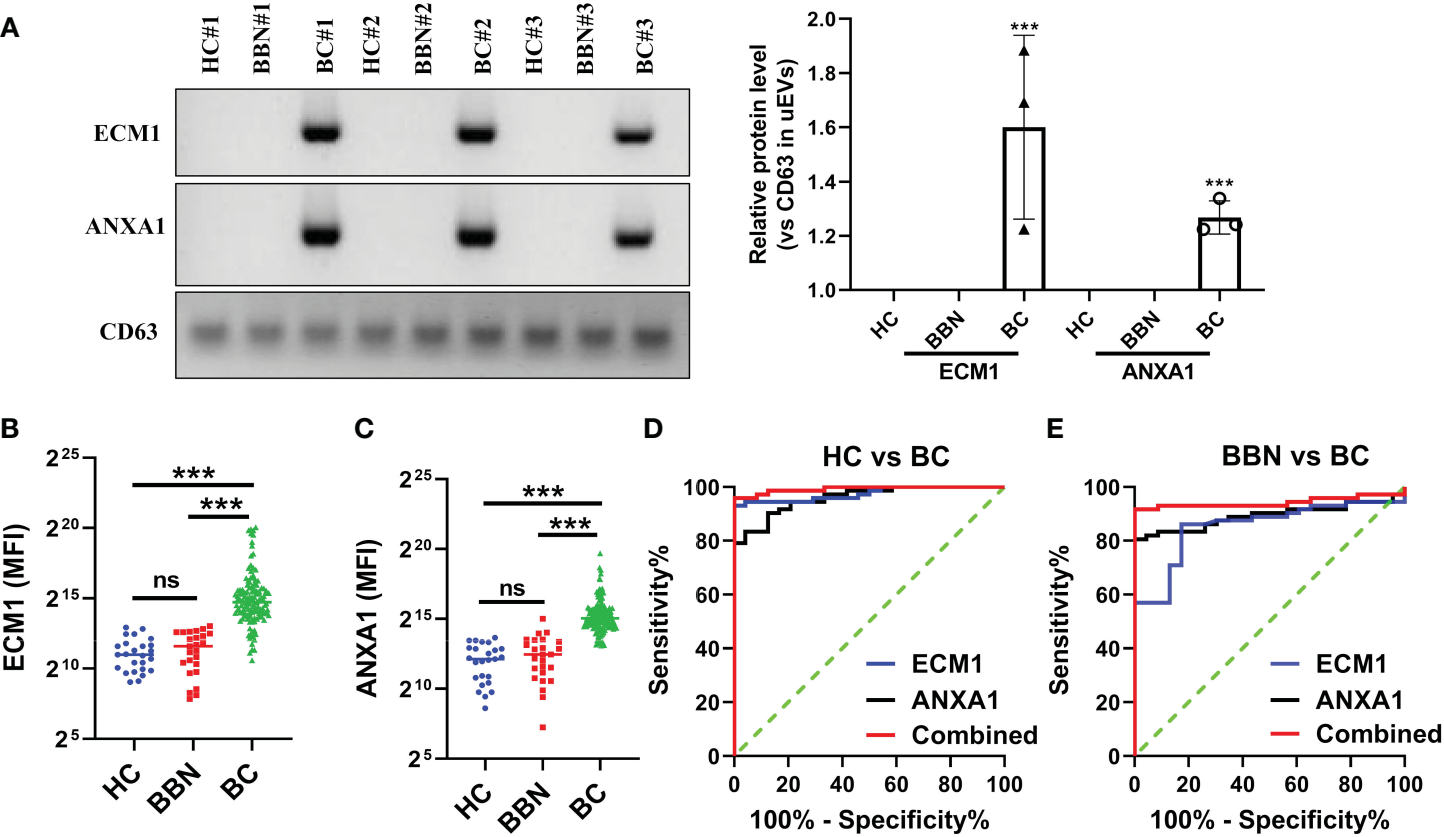
ECM1 and ANXA1 proteins contained in uEVs used as markers for diagnosing BC. **(A)** Western blotting analysis of expression levels of ECM1 and ANXA1 proteins in uEVs from patients with breast cancer (BC), healthy controls (HC) and patients with benign breast nodules (BBN), total protein in the isolated uEV were used as loading controls. Data from three independent experiments. **(B, C)** CLIA of expression levels of ECM1 and ANXA1 proteins in uEVs from patients with breast cancer (BC, n = 128), healthy controls (HC, n = 25) and patients with benign breast nodules (BBN, n = 25). **(D)** ROC curves analysis of ECM1, ANXA1 and their combination in uEVs to distinguish breast cancer (BC) from healthy control (HC). **(E)** ROC curves analysis of ECM1, ANXA1 and their combination in uEVs to distinguish breast cancer (BC) from benign breast nodules (BBN). Data from three independent experiments (n = 3) were presented as mean ± SD. ns: *p* > 0.05; ***p < 0.01.

To investigate whether ECM1 and ANXA1 in uEVs could be used as potential biomarkers for the diagnosis of breast cancer. Urine samples were collected from 25 HCs, 25 patients with BBNs and 128 breast cancer patients. Western blot analysis revealed that ECM1 and ANXA1 were highly expressed in the uEVs of breast cancer patients, but not in healthy controls or patients with benign nodules (**Figures 4A, B**). The results of the CLIA-confirmed quantitative analysis indicate that the protein levels of ECM1 and ANXA1 in the uEVs of BCs were significantly higher than those in HCs and BBNs (**Figures 4B, C**). Furthermore, ROC curve analysis demonstrated that the protein levels of ECM1 and ANXA1 in uEVs could effectively differentiate between breast cancer patients and healthy controls or patients with benign breast nodules (**Figures 4D, E**; **Table 2**). The results of the binary logistic regression analysis indicated that the combined model, comprising a logistic regression model with two proteins, exhibited the highest discriminatory power in distinguishing between breast cancer patients and healthy controls or patients with benign breast nodules (**Figures 4D, E**; **Table 2**).

**Table 2:** The diagnostic value of ECM1 and ANXA1 in uEVs in BC with HC or BBN.

		AUC	95% CI	p	Sensitivity%	Specificity%
**ECM1**	BC vs HC	0.9583	0.9233 to 0.9933	<0.0001	79.17	100
	BC vs BBN	0.8919	0.8273 to 0.9565	<0.0001	80.56	100
**ANXA1**	BC vs HC	0.9745	0.9477 to 1.000	<0.0001	93.06	100
	BC vs BBN	0.8536	0.7748 to 0.9323	<0.0001	86.11	82.61
**Combined**	BC vs HC	0.9925	0.9809 to 1.000	<0.0001	95.83	100
	BC vs BBN	0.9426	0.8932 to 0.9921	<0.0001	91.67	100

**Figure 5:**
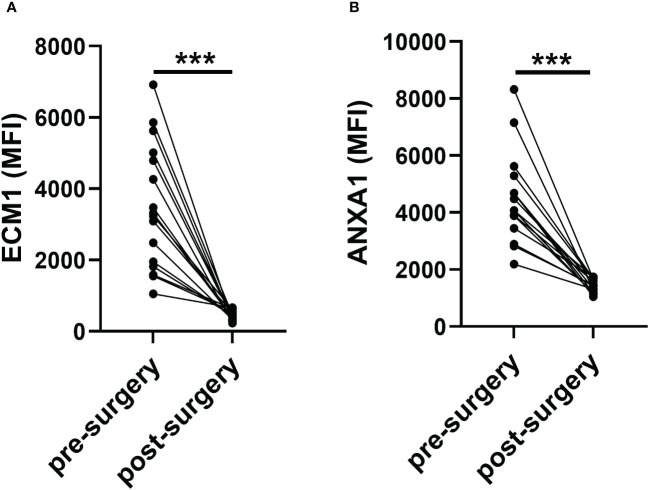
Revert level of ECM1 and ANXA1 in uEVs of breast cancer patients pre- or one week after post-surgery (n = 16). **(A, B)** CLIA of expression levels of ECM1 and ANXA1 proteins in uEVs breast cancer patients pre- or one week after post-surgery. Data from three independent experiments (n = 3) were presented as mean ± SD. ns: *p* > 0.05; ***p < 0.01.

Furthermore, we also evaluated the changes of ECM1 and ANXA1 levels in the uEVs from breast cancer patients before and after surgery using CLIA. The findings indicated a significant decrease in the levels of ECM1 and ANXA1 in the uEVs of breast cancer patients after surgery, compared to before surgery (**Figures 5A, B**). These results suggested that the levels of ECM1 and ANXA1 in uEVs could serve as diagnostic markers for breast cancer patients.

**Figure 6:**
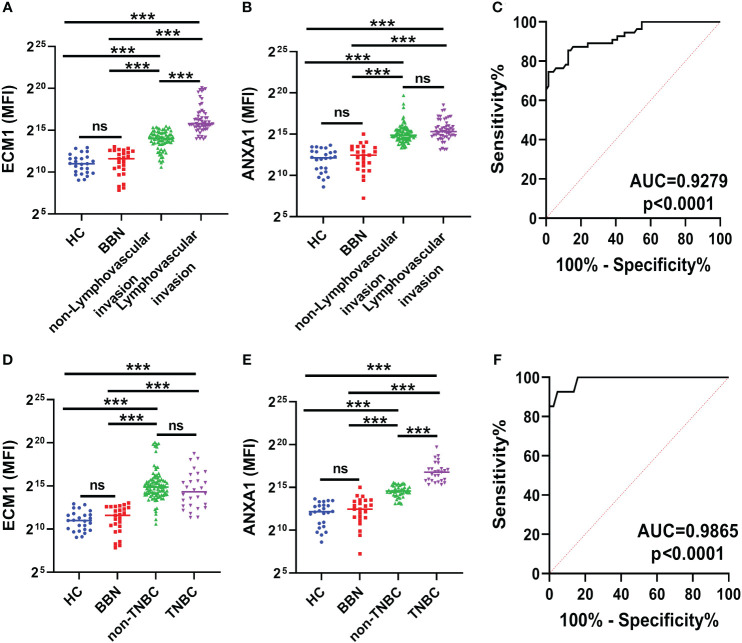
ECM1 and ANXA1 in uEVs of breast cancer patients with or without LVI and of non-TNBC or TNBC. **(A, B)** CLIA of expression levels of ECM1 and ANXA1 proteins in uEVs from patients with (n = 55) or without (n = 73) LVI. **(C)** ROC curves analysis of ECM1 and their combination in uEVs to distinguish breast cancer with LIV from breast cancer without LIV. **(D, E)** CLIA of expression levels of ECM1 and ANXA1 proteins in uEVs from patients of non-TNBC (n = 28) or TNBC (n = 100). **(F)** ROC curves analysis of ANXA1 and their combination in uEVs to distinguish non-TNBC breast cancer from TNBC breast cancer. Data from three independent experiments (n = 3) were presented as mean ± SD. ns: *p* > 0.05; ***p < 0.01.

The protein levels of ECM1 and ANXA1 in the uEVs of breast cancer patients with and without LVI were also compared. The results indicated that ECM1 levels were significantly higher in the uEVs of BC patients with LVI compared to those without LVI, while ANXA1 levels did not show significant differences (**Figures 6A, B**). The area under the ROC curve (AUC) for ECM1 in uEVs was 0.9279, with a sensitivity of 74.55% and a specificity of 98.59% (**Figure 6C**; **Table 3**). These findings suggest that ECM1 in uEVs may serve as a potential biomarker for predicting LVI in breast cancer patients. It is noteworthy that the protein levels of ANXA1 were significantly higher in the uEVs of TNBC patients compared to other breast cancer patients, while ECM1 levels did not show a significant difference (**Figures 6D, E**). The AUC for ANXA1 in uEVs was 0.9865 with a sensitivity of 92.59% and a specificity of 95.45% (**Figure 6F**; **Table 3**). These findings indicate that ANXA1 in uEVs may serve as a potential biomarker for the prediction of TNBC in breast cancer patients.

**Table 3:** The diagnostic value of ECM1 and ANXA1 in uEVs in prognosis and classification of BC.

		AUC	95% CI	p	Sensitivity%	Specificity%
**ECM1**	non-LVI vs LVI	0.9279	0.8830 to 0.9728	<0.0001	74.55	98.59
**ANXA1**	non-TNBC vs TNBC	0.9865	0.9678 to 1.000	<0.0001	92.59	95.45

### The protein level of ECM1 and ANXA1 in the uEVs upregulated in MMTV-PyMT transgenic mice

**Figure 7:**
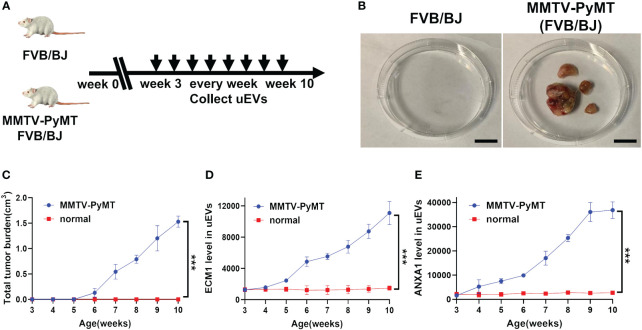
The protein level of ECM1 and ANXA1 in uEVs of MMTV-PyMT transgenic mice. **(A)** Flow chart of the experimental design. FVB/N transgenic mice expressing the PyMT oncogene under the control of the MMTV LTR promoter (FVB/N-TgN (MMTV-PyMT) 634-Mul) were used. After 3 weeks of age, the uEVs of FVB/BJ and MMTV-PyMT FVB/BJ mice were collected weekly. **(B)** Image record of tumor morphology of mice FVB/BJ and MMTV-PyMT FVB/BJ mice. Scale bars, 1 cm. **(C)** Record the total tumor burden(cm^3^) of MMTV-PyMT FVB/BJ mice weekly (n = 3 mice per group). **(D, E)** CLIA of expression levels of ECM1 and ANXA1 proteins in uEVs of MMTV-PyMT FVB/BJ mice every week (n = 3 mice per group). Data from three independent experiments (n = 3) were presented as mean ± SD. ***p < 0.01.

Urine samples were collected from MMTV-PyMT transgenic mice on a weekly basis from week 3 to 10 (**Figure 7A**). Mammary tumors became visible at week 6 and gradually increased in size and number (**Figures 7B, C**). The levels of ECM1 and ANXA1 in the uEVs were quantitatively measured using CLIA. The levels of ECM1 and ANXA1 in uEVs were found to be significantly elevated in MMTV-PyMT transgenic mice from week 5 compared to normal mice, with an increase in tumor size (**Figures 7D, E**). This suggests that ECM1 and ANXA1 in uEVs could be potential biomarkers for early diagnosis of breast cancer.

## Discussion

Breast cancer is a significant global health challenge with severe consequences (27). Despite efforts in cancer detection and treatment, breast cancer mortality rates have not improved significantly in recent decades. Patients diagnosed with advanced-stage breast cancer often have limited treatment options and a poor prognosis. Therefore, early diagnosis is critical for effective treatment and improved patient survival. Although blood is frequently studied for biomarkers, urine presents several advantages, such as easy and non-invasive sampling, less complex and lower dynamic range of proteins. We have analyzed human uEVs and identified over 3000 proteins in healthy controls and breast cancer patients. It has been confirmed that uEV profiling has potential as a diagnostic tool for breast cancer. This discovery provides a valuable resource for further research on urinary biomarkers. Furthermore, proteins associated with cancer progression in the uEVs of breast cancer patients were identified using the PANTHER database. In conclusion, the uEV marker identified in this study has the potential to benefit a large population and could be used in the future for early clinical diagnosis of breast cancer after further validation with larger sample sizes in a multicenter setting.

The uEVs were found to contain ECM1 and ANXA1, which were validated by CLIA as effective markers for discriminating breast cancer patients from healthy controls or patients with benign breast nodules. Notably, ECM1 levels were significantly higher in the uEVs of BCs with LVI compared to those without LVI. ANXA1 levels were significantly higher in the uEVs of patients with TNBC compared to those without TNBC. ECM1 has previously been implicated in breast cancer metastasis and plays a critical role in its progression by activating cholesterol biosynthesis, inducing NOTCH-mediated endothelial feedback and regulating actin cytoskeletal architecture through alterations in S100A4 and Rho A, suggesting its utility as an adjuvant therapeutic agent (28–33). Our study found elevated levels of ECM1 in uEVs of breast cancer patients. Additionally, significantly higher levels of ECM1 were observed in the uEVs of patients with LVI compared to those without LVI. These findings indicate that ECM1 in uEVs could be a potential biomarker for predicting LVI in breast cancer patients. ANXA1 is a multifunctional protein that is involved in various physiological processes, including hormone secretion, epidermal growth factor receptor degradation, membrane transport, apoptosis and cell differentiation. It also plays a crucial role in the inflammatory response and is regulated by Calcium ion. ANXA1 binds to cell membrane phospholipids and can be phosphorylated by the epidermal growth factor receptor, TRPM7 channel kinase 1, protein kinase A and protein kinase C. Its expression has been linked to carcinogenesis and metastasis in various tumor types, including breast tumors. Studies have demonstrated that ECM1 has the capacity to activate Transforming growth factor β and ERK pathways, which are involved in the metastatic cascade and the immune landscape of TNBC tumors. The study presented here corroborates the elevated levels of ECM1 observed in uEVs of breast cancer patients. Significantly higher levels of ECM1 were identified in patients with triple-negative breast cancer compared to other subtypes. This indicates a potential correlation between ECM1 expression and the development and progression of this subtype of breast cancer.

Several studies have explored EV testing for the diagnosis and prognosis of breast cancer before (34). EVs from the blood of breast cancer patients have been analyzed for the abundance of known RNAs and proteins (34). Blood and urine are two frequently researched biomatrices for discovery of biomarkers of human diseases. However, urine has several advantages over blood. Firstly, it can be easily obtained in large volumes and sampled frequently and complete non-invasively. Secondly, urine is less complex and has a relatively lower dynamic range, thus those low abundant but functionally important proteins in the uEVs can be reliably measured (34). In many economically underdeveloped areas, such as the herdsmen in the pastoral areas of Inner Mongolia, even simple blood drawing is difficult to carry out smoothly, let alone the use of imaging and puncture to diagnose breast cancer. Consequently, the awareness rate of breast cancer in these areas is very low. Most patients will enter formal hospitals for diagnosis and treatment only when they have obvious symptoms. However, the optimal opportunity for treatment has often been missed, resulting in a significantly higher mortality rate caused by breast cancer. In this study, we developed a simple, cheap and effective method for isolating and quantitatively detecting specific proteins in uEVs for the diagnosis of breast cancer by WGA coupled with magnetic beads and CLIA. This enables high-risk groups to engage in routine breast cancer screening including economically underdeveloped areas.

This study has several limitations, as it included a small number of participants, even though the current data suggest the potential of ECM1 and ANXA1 in the uEVs to distinguish breast cancer patients. A detailed study with a larger cohort is necessary to establish ECM1 and ANXA1 in the uEVs as potential biomarkers for breast cancer before starting clinical application. Another potential limitation of the study is that follow-up data were not available. It is not possible to speculate whether ECM1 and ANXA1 in the uEVs are associated with the survival and cure rate of breast cancer. Furthermore, although our method is already relatively simple, CLIA based methods still require the use of a spectrophotometer. For medical institutions in economically underdeveloped areas, purchasing this equipment still represents a significant financial outlay. In future research, we will attempt to develop a method that can detect ECM1 and ANXA1 in the uEVs using test strips by combining WGA-coupled magnetic beads with immunochromatography. This will enable high-risk groups to monitor their risk of developing breast cancer at home or in the community through urine and test strips on a regular basis.

The goal of developing a non-invasive test for BC risk has been pursued for over 20 years. Our study indicates that urinary proteins in uEVs could potentially serve as an indicator of the presence of BC during screening. This could facilitate direct examination and pathology testing for a definitive diagnosis. The study validated the protein levels of ECM1 and ANXA1 in the uEVs of BCs using CLIA. The proteins were found to be significantly elevated, and could serve as specific and sensitive biomarkers for diagnosing breast cancer patients.

## Data availability statement

The datasets presented in this study can be found in online repositories. The names of the repository/repositories and accession number(s) can be found in the article/**Supplementary Material**.

## Ethics statement

The studies involving humans were approved by the ethics committee of the Peking University Cancer Hospital (Inner Mongolia Campus), Affiliated Cancer Hospital of Inner Mongolia Medical University. The studies were conducted in accordance with the local legislation and institutional requirements. Written informed consent for participation in this study was provided by the participants’ legal guardians/next of kin. The animal studies were approved by the Ethical Committee of Nanjing University. The studies were conducted in accordance with the local legislation and institutional requirements. Written informed consent was obtained from the owners for the participation of their animals in this study.

## Author contributions

HH: Data curation, Investigation, Writing – original draft. JW: Investigation, Writing – original draft. XA: Writing – original draft. SQ: Writing – original draft. MJ: Writing – original draft. KZ: Writing – original draft. JL: Writing – original draft. KZ: Writing – original draft. HL: Writing – original draft, Writing – review & editing.
