# Supplementary material for: ECM1 and ANXA1 in urinary extracellular vesicles serve as biomarkers for breast cancer
Source: Front Oncol. 2024 Jul 8;14:1408492. doi: 10.3389/fonc.2024.1408492 (PMC11260749; doi:10.3389/fonc.2024.1408492)
Supplement: Supplementary file 1 [file DataSheet_1.docx]

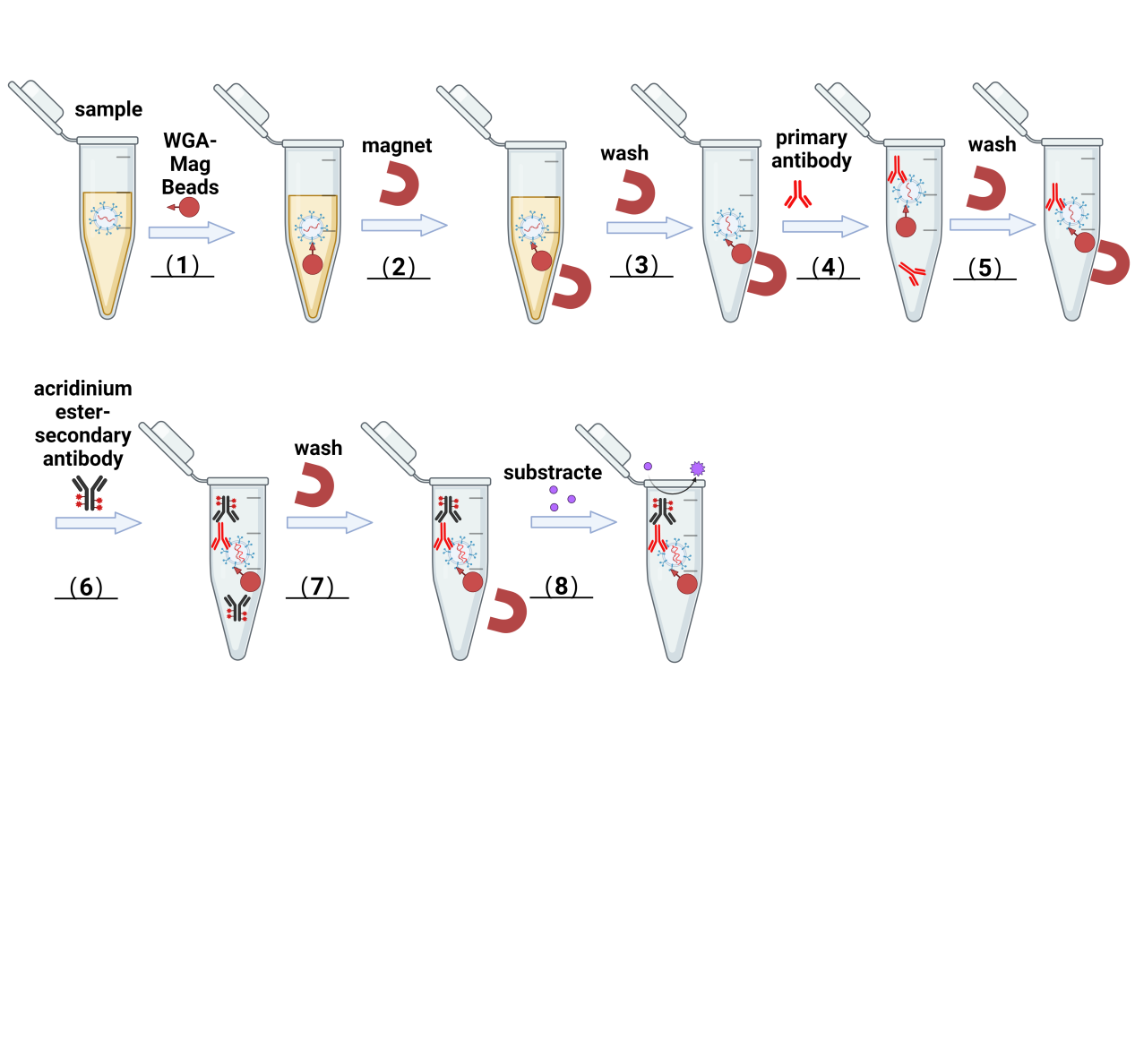


**Figure S1.** Flow chart of the method to capture extracellular vesicles in urine by WGA-coupled magnetic beads and detect the level of protein in the uEVs by CLIA. Since WGA binds to glycoproteins on the surface of extracellular vesicles in urine, the magnetic beads coupled with WGA can capture extracellular vesicles in the urine. Following incubation with an antibody targeting the specific protein marker and then acridinium ester labelled secondary antibody, the concentration of protein in the uEVs was determined.


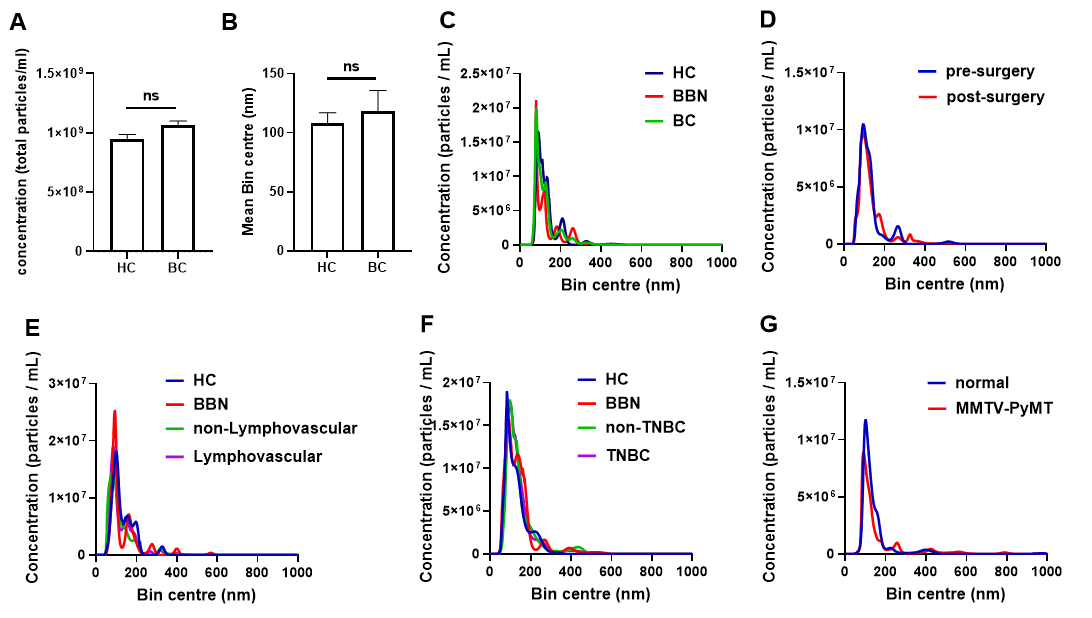


**Figure S2.** Quantification of uEVs isolated by WGA-coupled magnetic beads. (A) The total particles in urine of HC and BC patients. (B) The mean bin centre of the particles in urine of HC and BC patients. (C) NTA quantification of uEVs isolated from HC, BBN and BC urine; (D) NTA quantification of uEVs isolated from pre- or post-surgery urine; (E) NTA quantification of uEVs isolated from HC, BBN, non-Lymphovascular and Lymphovascular urine; (F) NTA quantification of uEVs isolated from HC, BBN, non-TNBC and TNBC urine; (G) NTA quantification of uEVs isolated from normal and MMTV-PyMT mouse urine;

**
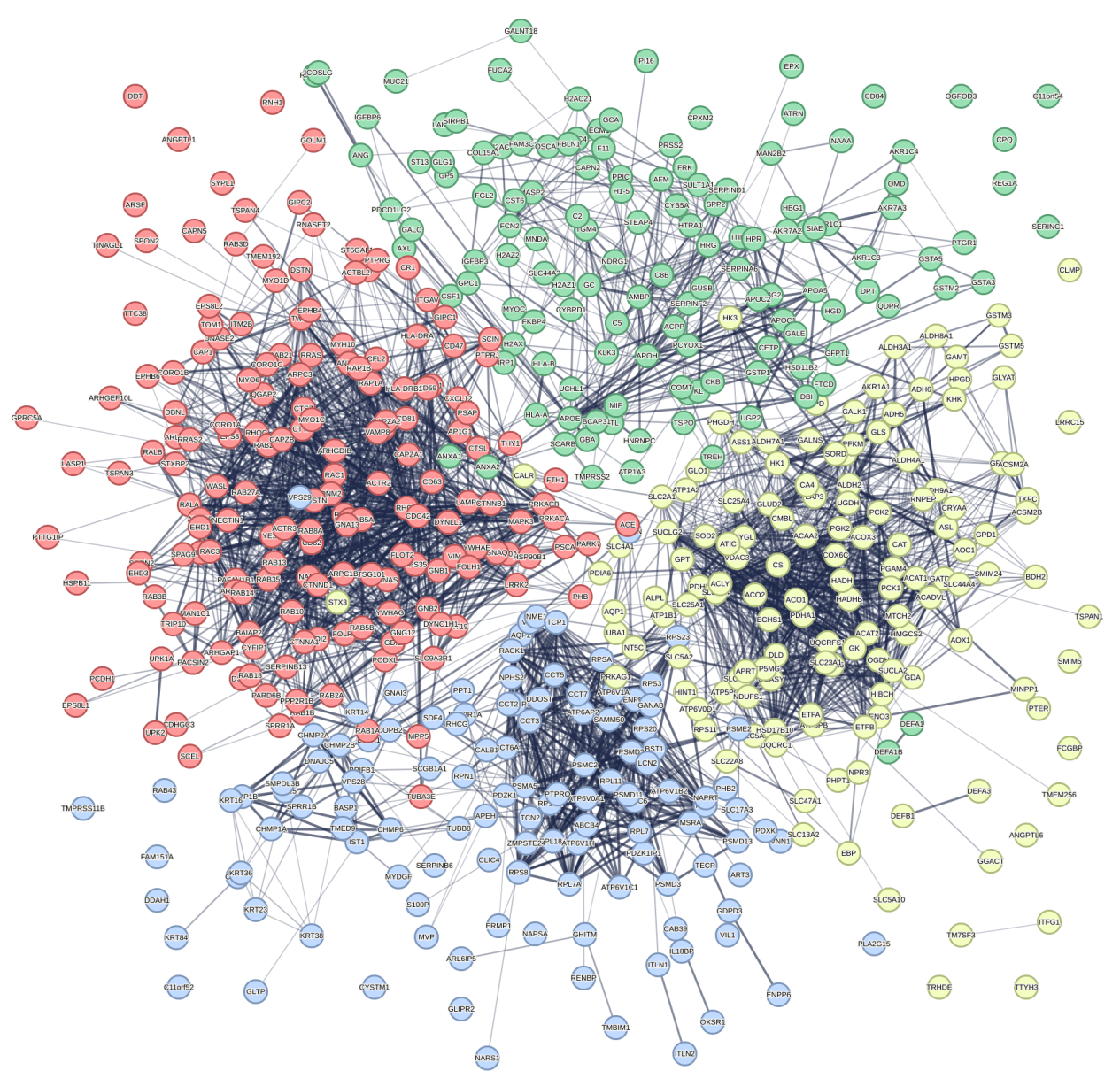
**

**Figure S3. The network analysis by STRING for the dysregulated proteins.**


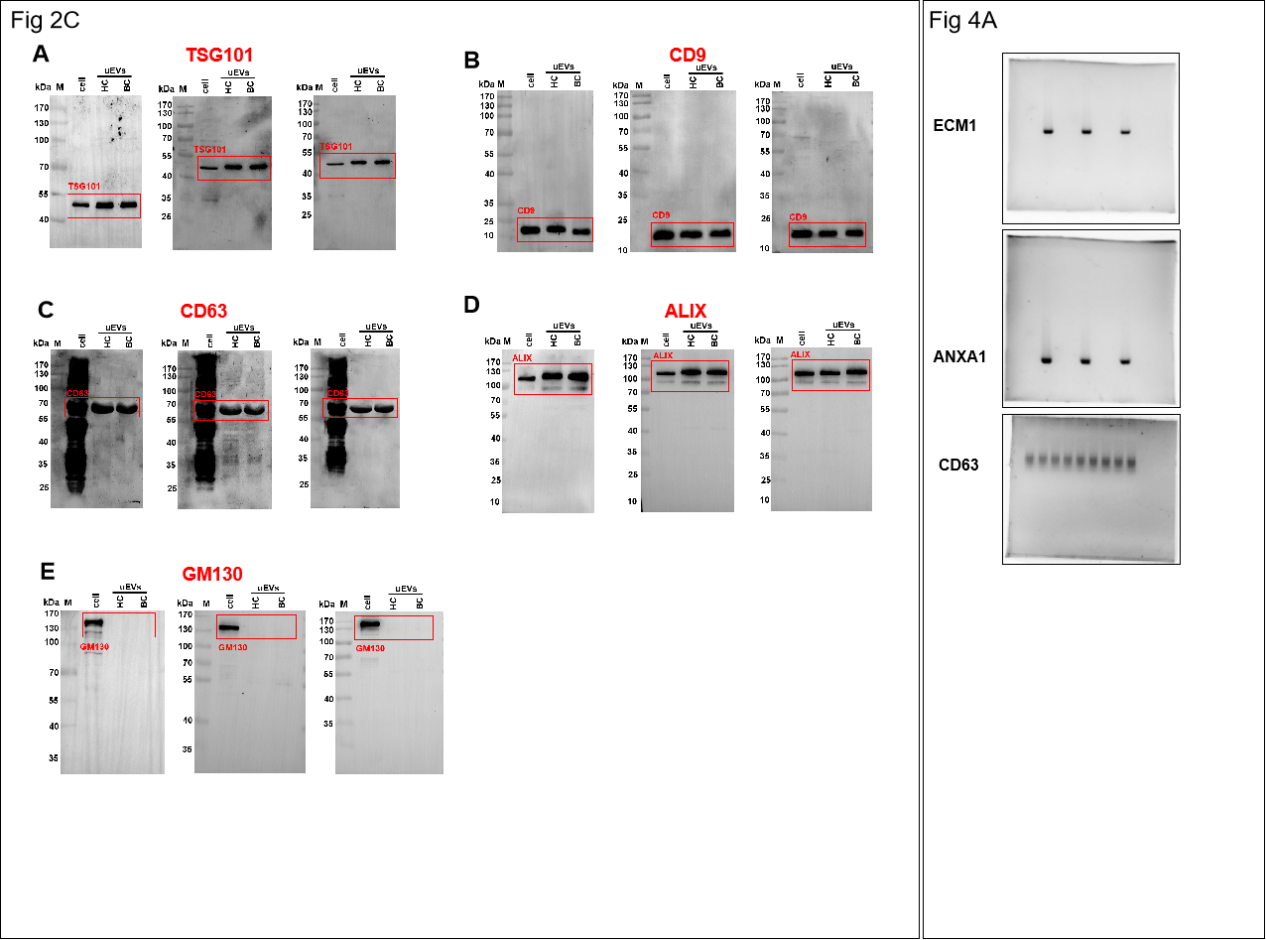


**Figure S4. Raw data for western blotting.**

**S1 Table** the dysregulted protein in the uEVs.

| **Gene** Symbol | **B**C(Mean) | **HC(Mean)** | **Fold change** | **Change** | **p** |
| --- | --- | --- | --- | --- | --- |
| CPQ | 1195.387694 | 479.9331923 | 2.490737696 | upregulated | 0.028418432 |
| KRT15 | 916.0504874 | 426.1783265 | 2.149453481 | upregulated | 0.001698481 |
| KRT19 | 520.9802154 | 239.4427591 | 2.175802757 | upregulated | 0.006584021 |
| KRT16 | 325.4084005 | 147.6337559 | 2.204159872 | upregulated | 0.007212551 |
| ECM1 | 322.4618343 | 26.02920926 | 12.38846063 | upregulated | 3.70E-08 |
| KRT14 | 317.6096673 | 151.3940535 | 2.097900545 | upregulated | 0.00183924 |
| ANXA2 | 305.8852115 | 106.6762047 | 2.867417457 | upregulated | 0.013675381 |
| GSTP1 | 242.1937079 | 59.87278816 | 4.04513829 | upregulated | 0.03365664 |
| ANXA1 | 129.524923 | 4.500677264 | 28.7789849 | upregulated | 7.30E-06 |
| IST1 | 118.048718 | 47.61775726 | 2.479090255 | upregulated | 0.000365085 |
| COL15A1 | 55.52260577 | 11.96566193 | 4.640161662 | upregulated | 0.045478699 |
| GUSB | 28.38497219 | 11.46849635 | 2.47503869 | upregulated | 0.000183459 |
|  |  |  |  |  |  |
| AMBP | 726.6188212 | 2138.862766 | 0.33972204 | downregulated | 0.006294071 |
| MASP2 | 565.4289995 | 1822.293424 | 0.310284278 | downregulated | 0.032886053 |
| ACPP | 98.76198807 | 505.8569313 | 0.195236997 | downregulated | 0.002963377 |
| ASS1 | 36.79792394 | 137.0622296 | 0.268476035 | downregulated | 0.003319176 |
| APOE | 38.01424679 | 129.9483316 | 0.29253355 | downregulated | 0.00913315 |
| AKR1A1 | 25.93678847 | 97.9925517 | 0.264681223 | downregulated | 0.021090743 |
| DEFA3 | 0.001 | 96.50715032 | 1.04E-05 | downregulated | 0.025933628 |
| DEFA1B | 0.001 | 96.50371753 | 1.04E-05 | downregulated | 0.025926741 |
| DEFA1 | 0.001 | 96.50371753 | 1.04E-05 | downregulated | 0.025926741 |
| NAPSA | 15.69118723 | 95.81343287 | 0.163768135 | downregulated | 0.005951357 |
| KLK3 | 0.001 | 83.27540556 | 1.20E-05 | downregulated | 0.005498416 |
| CHMP2A | 27.58497407 | 77.70169985 | 0.355011205 | downregulated | 0.021131894 |
| TMEM256 | 0.001 | 68.09972308 | 1.47E-05 | downregulated | 0.000547248 |
| THY1 | 10.80599877 | 65.91369082 | 0.163941643 | downregulated | 0.000412521 |
| CHMP1B | 5.834514361 | 64.48530537 | 0.0904782 | downregulated | 0.004269835 |
| VAMP8 | 0.001 | 64.00986232 | 1.56E-05 | downregulated | 0.000300857 |
| GSTM3 | 10.93246467 | 54.92981591 | 0.199026057 | downregulated | 0.001899018 |
| YWHAE | 15.81924424 | 49.8498407 | 0.31733791 | downregulated | 0.0003471 |
| GSTA3 | 2.0588602 | 49.16534752 | 0.041876246 | downregulated | 9.34E-05 |
| SORD | 6.147746093 | 49.04570543 | 0.125347287 | downregulated | 0.00206571 |
| AKR7A2 | 1.753702441 | 47.63635529 | 0.036814371 | downregulated | 0.002074218 |
| AQP2 | 8.976210859 | 47.04109091 | 0.190816384 | downregulated | 0.029133999 |
| CD59 | 0.001 | 46.58643844 | 2.15E-05 | downregulated | 7.30E-05 |
| CD63 | 11.94750575 | 46.01735215 | 0.259630448 | downregulated | 0.045776295 |
| UPK1A | 1.867179463 | 41.75211672 | 0.044720594 | downregulated | 0.01327639 |
| RAB10 | 7.203691021 | 41.06410722 | 0.175425487 | downregulated | 0.011406149 |
| CKB | 4.951776232 | 39.71257149 | 0.124690395 | downregulated | 0.000150139 |
| ACTBL2 | 15.12430568 | 36.18140009 | 0.418013279 | downregulated | 0.00494637 |
| PSAP | 3.600105104 | 35.20910524 | 0.102249264 | downregulated | 0.000233836 |
| RHOA | 5.886964583 | 34.43147283 | 0.170976264 | downregulated | 0.002502459 |
| FTL | 0.001 | 33.39113773 | 2.99E-05 | downregulated | 1.84E-06 |
| CXCL12 | 3.411455232 | 33.03749725 | 0.103260099 | downregulated | 0.002253108 |
| CMBL | 1.917285286 | 32.64772006 | 0.058726468 | downregulated | 0.000368181 |
| GNB2 | 7.875214813 | 32.64024679 | 0.241273139 | downregulated | 0.014034582 |
| CDC42 | 9.103722905 | 32.43064635 | 0.280713582 | downregulated | 0.00527758 |
| SYPL1 | 0.001 | 32.39122419 | 3.09E-05 | downregulated | 0.002938378 |
| SLC17A3 | 0.001 | 31.08234084 | 3.22E-05 | downregulated | 0.001856566 |
| PSCA | 0.001 | 29.0242361 | 3.45E-05 | downregulated | 0.000864389 |
| HBG2 | 0.001 | 28.81844825 | 3.47E-05 | downregulated | 0.01793217 |
| TSPAN1 | 10.32144764 | 28.61087833 | 0.360752561 | downregulated | 0.037524748 |
| MIF | 0.001 | 28.01428107 | 3.57E-05 | downregulated | 0.00139839 |
| YWHAG | 8.575406746 | 27.89443801 | 0.307423535 | downregulated | 0.014482996 |
| PODXL | 0.86258362 | 27.83605201 | 0.030988001 | downregulated | 0.000238353 |
| AQP1 | 0.001 | 27.20247122 | 3.68E-05 | downregulated | 0.002435983 |
| GNB1 | 6.574144049 | 26.59148093 | 0.247227451 | downregulated | 0.007557652 |
| AKR7A3 | 1.654498472 | 26.0165643 | 0.063594042 | downregulated | 0.000431287 |
| SERPINA6 | 0.001 | 26.01268069 | 3.84E-05 | downregulated | 0.000209771 |
| ACTR2 | 3.173133855 | 25.98583309 | 0.122110145 | downregulated | 0.001066306 |
| HBG1 | 0.001 | 25.87122467 | 3.87E-05 | downregulated | 0.011238274 |
| GGACT | 0.001 | 25.80299637 | 3.88E-05 | downregulated | 0.000310157 |
| APOA5 | 0.001 | 23.99842535 | 4.17E-05 | downregulated | 0.035215466 |
| S100A6 | 0.852536311 | 23.85602557 | 0.035736729 | downregulated | 0.009669182 |
| SLC44A2 | 2.595989361 | 23.4201787 | 0.110844131 | downregulated | 0.010718331 |
| UPK2 | 5.904531418 | 23.23845925 | 0.254084462 | downregulated | 0.031587382 |
| ARSF | 1.291742657 | 22.6664705 | 0.05698914 | downregulated | 0.0002186 |
| SOD2 | 0.500208973 | 22.65749565 | 0.022076975 | downregulated | 0.010642385 |
| MYH9 | 9.21226406 | 22.42516343 | 0.410800309 | downregulated | 0.047344974 |
| GC | 2.562558321 | 21.66442836 | 0.118284142 | downregulated | 0.001198308 |
| GLTP | 0.792015037 | 21.41899119 | 0.036977233 | downregulated | 0.000464245 |
| PHB2 | 1.644818463 | 21.31354151 | 0.077172462 | downregulated | 0.00134283 |
| FTCD | 3.632430959 | 21.0743112 | 0.172362974 | downregulated | 0.002006885 |
| RAB35 | 1.956036562 | 20.62497068 | 0.094838271 | downregulated | 0.003241411 |
| TMPRSS11B | 2.265099157 | 20.50295491 | 0.110476717 | downregulated | 0.016404306 |
| IGFBP3 | 0.001 | 20.28336829 | 4.93E-05 | downregulated | 0.000736112 |
| ITM2B | 3.134991739 | 20.21449055 | 0.155086359 | downregulated | 0.002259151 |
| SAMM50 | 1.554481033 | 20.11586064 | 0.077276387 | downregulated | 0.000234205 |
| PTER | 0.066787889 | 19.80675846 | 0.003371975 | downregulated | 0.001239536 |
| SPRR1B | 0.001 | 19.78055327 | 5.06E-05 | downregulated | 0.019384562 |
| DAK | 5.464099756 | 19.68277601 | 0.277608187 | downregulated | 0.040067645 |
| PCYOX1 | 1.343428745 | 19.49982938 | 0.068894385 | downregulated | 0.00313385 |
| HLA-A | 4.437990712 | 19.22754623 | 0.230814201 | downregulated | 0.034696575 |
| PDZK1 | 1.219892862 | 19.20446084 | 0.063521328 | downregulated | 0.000148183 |
| ACE | 1.648975274 | 18.88955142 | 0.087295629 | downregulated | 0.010901773 |
| RPS8 | 2.703473374 | 18.76951326 | 0.144035348 | downregulated | 0.000637991 |
| SPRR1A | 0.001 | 18.40332346 | 5.43E-05 | downregulated | 0.023135495 |
| CAPZB | 3.934585566 | 18.40236245 | 0.21380872 | downregulated | 0.016312643 |
| VDAC3 | 3.440000275 | 18.32207909 | 0.187751633 | downregulated | 0.012553933 |
| GSTA5 | 0.631417367 | 18.23468797 | 0.034627265 | downregulated | 0.000186278 |
| AOC1 | 3.544219874 | 17.80414472 | 0.199067123 | downregulated | 0.000257834 |
| RAP1B | 1.512970102 | 17.71247581 | 0.08541833 | downregulated | 0.000903245 |
| KHK | 4.586660506 | 17.64310462 | 0.259969014 | downregulated | 0.027966437 |
| ANXA7 | 4.901298256 | 17.37041385 | 0.282163586 | downregulated | 0.020887072 |
| DEFB1 | 0.001 | 17.11140708 | 5.84E-05 | downregulated | 0.039644948 |
| HPR | 0.001 | 16.88595346 | 5.92E-05 | downregulated | 0.006954698 |
| TCP1 | 1.197469473 | 16.73465398 | 0.071556273 | downregulated | 0.003969675 |
| CYSTM1 | 0.001 | 16.72360038 | 5.98E-05 | downregulated | 0.042945838 |
| GNG12 | 0.001 | 16.62777984 | 6.01E-05 | downregulated | 8.08E-06 |
| RAB8A | 0.421961637 | 16.5204802 | 0.02554173 | downregulated | 3.09E-05 |
| RAP1A | 1.46500675 | 16.4348859 | 0.089140062 | downregulated | 0.000626178 |
| PCK1 | 2.886033157 | 16.42065588 | 0.175756266 | downregulated | 0.004705359 |
| DNAJC5 | 0.001 | 16.14190795 | 6.20E-05 | downregulated | 0.023665384 |
| BDH2 | 0.532435388 | 16.1020288 | 0.033066354 | downregulated | 2.53E-05 |
| CLIC4 | 0.001 | 15.8666522 | 6.30E-05 | downregulated | 0.000480888 |
| ATP6V1A | 4.271461515 | 15.58174667 | 0.274132394 | downregulated | 0.021671474 |
| SMIM5 | 0.001 | 15.45051827 | 6.47E-05 | downregulated | 0.01253187 |
| EHD1 | 2.54241184 | 15.28150522 | 0.16637182 | downregulated | 0.001515879 |
| NT5C | 3.35794838 | 15.10423738 | 0.2223183 | downregulated | 0.005760331 |
| CETP | 5.401304111 | 14.91169016 | 0.362219444 | downregulated | 0.025432331 |
| ARPC1A | 1.699961757 | 14.66193871 | 0.115943859 | downregulated | 0.011460859 |
| VCP | 5.033438118 | 14.59493909 | 0.344875582 | downregulated | 0.044896237 |
| FTH1 | 0.001 | 14.50086393 | 6.90E-05 | downregulated | 2.43E-05 |
| LAMP1 | 2.872895387 | 14.22724829 | 0.201929096 | downregulated | 0.018205283 |
| NCSTN | 3.51420099 | 14.04485963 | 0.25021261 | downregulated | 0.005499962 |
| GPD1 | 3.753682552 | 13.7734535 | 0.272530237 | downregulated | 0.049352373 |
| PHPT1 | 0.001 | 13.72704216 | 7.28E-05 | downregulated | 0.00034445 |
| TSG101 | 5.428559182 | 13.40142302 | 0.40507334 | downregulated | 0.023716516 |
| RALB | 1.494232555 | 13.39996554 | 0.111510179 | downregulated | 0.000343149 |
| CTSZ | 0.001 | 13.11887521 | 7.62E-05 | downregulated | 0.000354769 |
| APRT | 2.527416409 | 12.9475268 | 0.195204571 | downregulated | 0.006118369 |
| ACO1 | 1.782306144 | 12.90216683 | 0.138140063 | downregulated | 0.001243523 |
| APOC1 | 1.360288167 | 12.67011253 | 0.107361964 | downregulated | 0.027884065 |
| RAB27A | 0.001 | 12.48713873 | 8.01E-05 | downregulated | 0.000184535 |
| PDZK1IP1 | 0.001 | 12.4690997 | 8.02E-05 | downregulated | 0.004561336 |
| HSP90B1 | 2.878351667 | 12.32955867 | 0.233451314 | downregulated | 0.002301968 |
| PTGR1 | 3.414079383 | 12.01482538 | 0.284155556 | downregulated | 0.035769376 |
| LOC101060439 | 0.476396912 | 12.01106765 | 0.039663161 | downregulated | 0.00176982 |
| SCARB2 | 0.362750813 | 11.74851925 | 0.030876301 | downregulated | 0.000136142 |
| ANG | 0.001 | 11.74366396 | 8.52E-05 | downregulated | 0.000540131 |
| RPS3A | 1.842769905 | 11.7304147 | 0.15709333 | downregulated | 0.001772847 |
| ACAT1 | 0.769216536 | 11.70029664 | 0.065743336 | downregulated | 0.002898462 |
| ATP1B1 | 1.010285589 | 11.66278333 | 0.086624741 | downregulated | 0.002195167 |
| UGP2 | 2.270635318 | 11.52853808 | 0.196957784 | downregulated | 0.015127085 |
| ALDH9A1 | 3.638604503 | 11.47152241 | 0.317185843 | downregulated | 0.026707583 |
| LYNX1 | 0.001 | 11.39892685 | 8.77E-05 | downregulated | 0.006396837 |
| MAL2 | 0.001 | 11.36067318 | 8.80E-05 | downregulated | 0.040287491 |
| EPS8L2 | 2.283575645 | 11.29163667 | 0.202236019 | downregulated | 0.003602904 |
| RAB21 | 0.410335152 | 11.11489207 | 0.036917601 | downregulated | 0.000377984 |
| ATP5L | 0.001 | 11.10842013 | 9.00E-05 | downregulated | 0.002289207 |
| SLC9A3R1 | 0.490708084 | 10.91736665 | 0.044947477 | downregulated | 0.00014276 |
| GPRC5A | 0.001 | 10.79196643 | 9.27E-05 | downregulated | 0.00486269 |
| HLA-DRA | 0.001 | 10.70057559 | 9.35E-05 | downregulated | 0.008538061 |
| ADH5 | 0.630042894 | 10.5905835 | 0.059490857 | downregulated | 0.001849288 |
| HPGD | 2.657748683 | 10.55999533 | 0.251680858 | downregulated | 0.015264961 |
| HRG | 3.765775708 | 10.3776134 | 0.362874927 | downregulated | 0.033300559 |
| RAB14 | 1.189974959 | 10.30077258 | 0.115522884 | downregulated | 0.000998594 |
| PLA2G15 | 3.45620919 | 10.12591463 | 0.341323161 | downregulated | 0.038629945 |
| KRT84 | 2.468300012 | 9.869219321 | 0.250100837 | downregulated | 0.005053057 |
| EPHB6 | 0.001 | 9.839082328 | 0.000101635 | downregulated | 0.008347273 |
| ACTR3 | 2.26129699 | 9.742298972 | 0.232111229 | downregulated | 0.005153525 |
| DDAH1 | 1.835670037 | 9.638205658 | 0.190457654 | downregulated | 0.014185612 |
| DPT | 0.001 | 9.585226172 | 0.000104327 | downregulated | 0.01312783 |
| DSTN | 0.800214402 | 9.526026527 | 0.084002958 | downregulated | 0.010079707 |
| GNA13 | 2.004987776 | 9.490800416 | 0.21125592 | downregulated | 0.008223893 |
| RALA | 1.230449981 | 9.453200961 | 0.130162258 | downregulated | 0.004775097 |
| FGL2 | 1.449315635 | 9.36360132 | 0.154781861 | downregulated | 0.005944582 |
| LAP3 | 1.780899526 | 9.350523212 | 0.19045988 | downregulated | 0.001675565 |
| CCT2 | 1.134133095 | 9.28864731 | 0.122098844 | downregulated | 0.00017863 |
| CR1 | 0.093320337 | 9.243625859 | 0.010095642 | downregulated | 0.000448127 |
| ATP6AP2 | 1.288107848 | 9.223746793 | 0.139651259 | downregulated | 0.002850253 |
| SLC25A4 | 2.230192172 | 9.16100723 | 0.243443992 | downregulated | 0.030755217 |
| RAC1 | 1.835613678 | 9.109341432 | 0.201508934 | downregulated | 0.007180414 |
| CAP1 | 2.110760587 | 9.037393073 | 0.233558568 | downregulated | 0.030502076 |
| RHCG | 0.79552663 | 8.940473706 | 0.088980367 | downregulated | 0.000536322 |
| SPP2 | 0.001 | 8.859964883 | 0.000112867 | downregulated | 0.004284136 |
| MVP | 2.005447524 | 8.772937937 | 0.228594747 | downregulated | 0.006627483 |
| FAM151A | 4.034126567 | 8.678754388 | 0.46482783 | downregulated | 0.025634435 |
| RPS3 | 1.637415091 | 8.642618334 | 0.189458221 | downregulated | 0.023750764 |
| PPP2R1A | 2.388512233 | 8.638724942 | 0.276488978 | downregulated | 0.019518442 |
| ALDH4A1 | 0.580005618 | 8.51864803 | 0.068086581 | downregulated | 0.00015359 |
| GNAS | 1.344212396 | 8.475755197 | 0.158595 | downregulated | 0.003104906 |
| NAPA | 1.881168341 | 8.384432567 | 0.224364419 | downregulated | 0.033135526 |
| GDI2 | 2.437885703 | 8.360387879 | 0.291599593 | downregulated | 0.013961564 |
| APOC2 | 0.001 | 8.327841373 | 0.000120079 | downregulated | 0.000317247 |
| MYOC | 0.001 | 8.301845608 | 0.000120455 | downregulated | 0.001400052 |
| GDA | 0.643939864 | 8.216629464 | 0.078370318 | downregulated | 0.002306699 |
| RPS9 | 1.887100189 | 8.17755409 | 0.230765846 | downregulated | 0.005666864 |
| C19orf77 | 0.610683659 | 8.021396178 | 0.076131841 | downregulated | 0.003132867 |
| RAB1B | 1.229815207 | 7.99484258 | 0.153826069 | downregulated | 0.00665618 |
| RAB27B | 0.001 | 7.861259005 | 0.000127206 | downregulated | 0.000541346 |
| SIAE | 0.740040228 | 7.779360677 | 0.095128669 | downregulated | 0.009169354 |
| GALK1 | 0.247131595 | 7.73137797 | 0.031964754 | downregulated | 0.000854705 |
| GALNS | 0.593654409 | 7.689885443 | 0.077199383 | downregulated | 0.000386001 |
| GANAB | 1.052071326 | 7.686649021 | 0.136869958 | downregulated | 0.001842626 |
| PGK2 | 2.222693356 | 7.660184883 | 0.290161842 | downregulated | 0.004197266 |
| SLC44A4 | 0.082457102 | 7.656618984 | 0.010769388 | downregulated | 0.002193519 |
| MYO6 | 1.624453308 | 7.473909235 | 0.217349884 | downregulated | 0.013835048 |
| CA4 | 0.575911833 | 7.462354467 | 0.07717562 | downregulated | 0.000319279 |
| GNB2L1 | 1.555963118 | 7.418010777 | 0.209754766 | downregulated | 0.0020869 |
| ST13 | 0.230385459 | 7.308420931 | 0.031523288 | downregulated | 0.01570427 |
| CAPZA2 | 1.784709341 | 7.254701578 | 0.246007271 | downregulated | 0.034092 |
| QDPR | 0.001 | 7.141206359 | 0.000140032 | downregulated | 0.000408224 |
| CCT7 | 0.531259422 | 7.135842771 | 0.074449429 | downregulated | 0.000893274 |
| PHGDH | 2.737454291 | 6.975364551 | 0.392446054 | downregulated | 0.008609747 |
| CST6 | 0.001 | 6.974754512 | 0.000143374 | downregulated | 0.003997013 |
| CALR | 2.286248638 | 6.951639738 | 0.328879045 | downregulated | 0.030966818 |
| CORO1B | 0.246473088 | 6.948271542 | 0.035472576 | downregulated | 0.000425728 |
| FUCA2 | 2.376718618 | 6.939868617 | 0.342473143 | downregulated | 0.025230383 |
| CCT3 | 0.712280911 | 6.899631183 | 0.103234636 | downregulated | 0.000471099 |
| TWF1 | 1.970128998 | 6.885283081 | 0.286136238 | downregulated | 0.015537236 |
| NDUFS1 | 0.104041706 | 6.865193652 | 0.015154956 | downregulated | 0.006293259 |
| GDPD3 | 0.708211829 | 6.801205979 | 0.104130331 | downregulated | 0.001324303 |
| PEPD | 0.714456887 | 6.783631222 | 0.105320714 | downregulated | 0.001612332 |
| OGFOD3 | 0.001 | 6.649447543 | 0.000150388 | downregulated | 0.00034777 |
| ITIH1 | 0.696311032 | 6.55354282 | 0.106249559 | downregulated | 0.007915493 |
| CCT5 | 1.043998279 | 6.531054453 | 0.159851412 | downregulated | 0.004728777 |
| RHOC | 0.001 | 6.47725419 | 0.000154386 | downregulated | 0.000210341 |
| FCN2 | 0.449050062 | 6.399465597 | 0.070169931 | downregulated | 0.00621039 |
| HADHB | 0.161901225 | 6.391479967 | 0.025330788 | downregulated | 0.003860445 |
| MAN2A1 | 1.069246666 | 6.340463797 | 0.168638557 | downregulated | 0.003473034 |
| PDXK | 0.185053897 | 6.310141438 | 0.029326426 | downregulated | 0.004833727 |
| STX3 | 0.040700889 | 6.254721249 | 0.006507227 | downregulated | 0.001030341 |
| UCHL1 | 0.001 | 6.230625221 | 0.000160498 | downregulated | 0.000203022 |
| ATP1A3 | 2.410740968 | 6.23040929 | 0.386931397 | downregulated | 0.025842914 |
| RAB1A | 1.572117061 | 6.200065579 | 0.253564586 | downregulated | 0.006014139 |
| PTTG1IP | 0.001 | 6.168700315 | 0.000162109 | downregulated | 0.001251677 |
| NDRG1 | 1.999580563 | 6.153903934 | 0.324928791 | downregulated | 0.026095194 |
| BASP1 | 0.049945342 | 6.070315435 | 0.0082278 | downregulated | 0.001265811 |
| GLYAT | 0.001 | 6.058710741 | 0.000165052 | downregulated | 0.000483017 |
| RRAS2 | 1.43692359 | 6.049000782 | 0.237547265 | downregulated | 0.030394657 |
| VPS28 | 2.36241725 | 6.036148165 | 0.391378274 | downregulated | 0.042950252 |
| ACO2 | 0.836124426 | 5.954016682 | 0.140430313 | downregulated | 0.002949434 |
| NPHS2 | 0.978093721 | 5.873319332 | 0.166531678 | downregulated | 0.021765849 |
| UBA1 | 1.791546596 | 5.782938979 | 0.309798634 | downregulated | 0.036992536 |
| SUCLG2 | 1.266650748 | 5.752588342 | 0.22018797 | downregulated | 0.019163787 |
| CHMP2B | 0.679897964 | 5.737065905 | 0.118509701 | downregulated | 0.00099935 |
| PRKAG1 | 0.001 | 5.718519432 | 0.00017487 | downregulated | 0.048591342 |
| TRHDE | 0.341980712 | 5.680869745 | 0.060198654 | downregulated | 0.000457646 |
| PI16 | 0.001 | 5.62915485 | 0.000177647 | downregulated | 0.004668592 |
| VIL1 | 0.001 | 5.548480532 | 0.00018023 | downregulated | 7.24E-07 |
| CRYAA | 0.001 | 5.504552305 | 0.000181668 | downregulated | 0.000261891 |
| ETFB | 0.472614565 | 5.450774151 | 0.086705953 | downregulated | 0.000591121 |
| PCK2 | 0.321452243 | 5.443569015 | 0.059051744 | downregulated | 0.000435112 |
| PTPRO | 0.033633689 | 5.403182102 | 0.006224793 | downregulated | 0.001768575 |
| STXBP2 | 0.335036612 | 5.389944129 | 0.06215957 | downregulated | 0.00015985 |
| KL | 0.154653247 | 5.381787385 | 0.02873641 | downregulated | 0.014335055 |
| APOH | 0.001 | 5.300642961 | 0.000188656 | downregulated | 0.000852336 |
| MYO1C | 0.408103362 | 5.290012378 | 0.077146013 | downregulated | 0.00088871 |
| SERPINF2 | 0.276326762 | 5.209844019 | 0.053039354 | downregulated | 0.000137778 |
| CYB5A | 0.190606494 | 5.153947048 | 0.036982626 | downregulated | 0.002321303 |
| ATP1A2 | 2.052660071 | 5.129484521 | 0.400168879 | downregulated | 0.040221477 |
| ARPC3 | 1.0375502 | 5.11056001 | 0.203020843 | downregulated | 0.014279529 |
| PHB | 0.783362296 | 5.076574324 | 0.154309234 | downregulated | 0.007175142 |
| RAB13 | 0.763641304 | 5.038482412 | 0.151561768 | downregulated | 0.013685726 |
| CPXM2 | 0.48294189 | 5.036201355 | 0.095894079 | downregulated | 0.02527212 |
| SCGB1A1 | 0.001 | 5.026530282 | 0.000198944 | downregulated | 0.00013211 |
| ITLN1 | 0.219585957 | 5.014509538 | 0.043790117 | downregulated | 0.023427952 |
| SLC6A19 | 0.499348468 | 4.972748573 | 0.100416995 | downregulated | 0.010705353 |
| AKR1C3 | 0.464677294 | 4.96469816 | 0.093596283 | downregulated | 0.001417614 |
| GNAI3 | 1.155974206 | 4.956042557 | 0.233245416 | downregulated | 0.00400575 |
| HK1 | 0.526273078 | 4.932735384 | 0.106689907 | downregulated | 0.041409007 |
| ACAA2 | 0.551858948 | 4.907738674 | 0.112446686 | downregulated | 0.002877667 |
| PPIC | 0.74519671 | 4.899178197 | 0.152106472 | downregulated | 0.001445258 |
| MYH10 | 0.282357218 | 4.859646495 | 0.058102419 | downregulated | 0.001923055 |
| GNAI1 | 1.224381181 | 4.826940912 | 0.253655722 | downregulated | 0.028965617 |
| SERPINB13 | 1.589887665 | 4.81378846 | 0.330277842 | downregulated | 0.044629629 |
| TTYH3 | 0.001 | 4.813492904 | 0.000207749 | downregulated | 0.000281195 |
| KRT36 | 0.001 | 4.804126031 | 0.000208154 | downregulated | 0.044239828 |
| CD81 | 0.001 | 4.77345389 | 0.000209492 | downregulated | 0.000877204 |
| TMPRSS2 | 0.129635595 | 4.767711958 | 0.027190316 | downregulated | 0.000376085 |
| FLOT2 | 0.485852321 | 4.757207682 | 0.102129727 | downregulated | 0.002767183 |
| ATP5F1 | 0.039152123 | 4.751063597 | 0.008240707 | downregulated | 0.000817981 |
| ATRN | 0.553205266 | 4.734366336 | 0.116848851 | downregulated | 0.001874043 |
| ALDH2 | 0.567759599 | 4.672696658 | 0.121505769 | downregulated | 0.000181726 |
| EHD3 | 1.101074032 | 4.668560008 | 0.235848748 | downregulated | 0.017219714 |
| SDF4 | 0.001 | 4.661562818 | 0.00021452 | downregulated | 0.005132488 |
| ECHS1 | 0.320397248 | 4.633212287 | 0.069152292 | downregulated | 0.001692947 |
| SLC4A1 | 1.252350529 | 4.57703021 | 0.2736164 | downregulated | 0.023175599 |
| COMT | 0.742572976 | 4.536846906 | 0.163676005 | downregulated | 0.004491279 |
| DSG3 | 0.491448425 | 4.515672814 | 0.108831717 | downregulated | 0.029234984 |
| RAB5B | 1.090481093 | 4.507693719 | 0.241915525 | downregulated | 0.025310536 |
| NARS | 0.314114448 | 4.431293839 | 0.070885493 | downregulated | 0.00026402 |
| RNASE4 | 0.001 | 4.399488029 | 0.000227299 | downregulated | 6.66E-06 |
| HINT1 | 0.167332141 | 4.37447903 | 0.038251901 | downregulated | 0.000457777 |
| TUBA3E | 1.469114818 | 4.364182916 | 0.336629982 | downregulated | 0.041968111 |
| DBI | 0.001 | 4.334527068 | 0.000230706 | downregulated | 5.40E-05 |
| ENO3 | 0.4723557 | 4.313411785 | 0.109508603 | downregulated | 0.002503367 |
| S100P | 0.001 | 4.299927782 | 0.000232562 | downregulated | 0.009201808 |
| RAB5A | 1.081371051 | 4.255647649 | 0.254102581 | downregulated | 0.029985866 |
| YES1 | 0.026299057 | 4.234719747 | 0.006210342 | downregulated | 0.029323457 |
| ACAT2 | 0.081123371 | 4.221114151 | 0.019218474 | downregulated | 0.000979604 |
| RNASET2 | 0.001 | 4.179997823 | 0.000239235 | downregulated | 0.009783203 |
| HGD | 0.242972341 | 4.165167902 | 0.058334345 | downregulated | 2.24E-05 |
| CYBRD1 | 0.001 | 4.143543836 | 0.000241339 | downregulated | 0.001272286 |
| ATP6V1H | 0.636147584 | 4.131833103 | 0.153962556 | downregulated | 0.004522509 |
| RAB2A | 0.709376119 | 4.126789807 | 0.171895384 | downregulated | 0.003952548 |
| UGDH | 0.336271468 | 4.112377948 | 0.081770565 | downregulated | 0.005599194 |
| ETFA | 0.419509977 | 4.058781273 | 0.103358607 | downregulated | 0.006425839 |
| CAPN5 | 0.001 | 4.02275821 | 0.000248586 | downregulated | 0.008086374 |
| FOLR1 | 0.001 | 4.018140667 | 0.000248871 | downregulated | 4.05E-05 |
| GLO1 | 1.079150603 | 3.968985609 | 0.271895822 | downregulated | 0.00829792 |
| PAFAH1B1 | 0.001 | 3.946236119 | 0.000253406 | downregulated | 0.016331369 |
| GK | 0.001 | 3.922265001 | 0.000254955 | downregulated | 0.001348493 |
| GNAQ | 0.676933813 | 3.91661088 | 0.172836627 | downregulated | 0.018221255 |
| HPD | 0.329374631 | 3.915993308 | 0.084110111 | downregulated | 0.001633298 |
| C11orf54 | 0.001 | 3.915568696 | 0.000255391 | downregulated | 0.000227239 |
| ENPP6 | 0.272575842 | 3.903546592 | 0.069827741 | downregulated | 0.002233252 |
| GIPC2 | 0.001 | 3.902403322 | 0.000256252 | downregulated | 0.004163198 |
| SCEL | 1.242483861 | 3.86779488 | 0.321238302 | downregulated | 0.000277081 |
| OXSR1 | 0.269208367 | 3.867024046 | 0.069616419 | downregulated | 0.00017938 |
| ARHGEF10L | 0.101725988 | 3.812856616 | 0.026679731 | downregulated | 1.51E-05 |
| ACADVL | 0.133067337 | 3.809633081 | 0.034929174 | downregulated | 0.002755796 |
| TM7SF3 | 0.001 | 3.798863799 | 0.000263237 | downregulated | 0.003929658 |
| PDCD1LG2 | 0.001 | 3.792696071 | 0.000263665 | downregulated | 7.07E-05 |
| RPL18A | 0.826064231 | 3.744590685 | 0.22060201 | downregulated | 0.007322212 |
| TUBB8 | 1.334148786 | 3.725251681 | 0.35813655 | downregulated | 0.041138899 |
| RRAS | 0.333962044 | 3.673452795 | 0.0909123 | downregulated | 0.009332265 |
| COX6C | 0.001 | 3.650847015 | 0.000273909 | downregulated | 0.01745582 |
| ACLY | 0.161291382 | 3.647570998 | 0.044218847 | downregulated | 0.000141463 |
| MPP5 | 0.001 | 3.630291884 | 0.00027546 | downregulated | 0.001250701 |
| SERPINB6 | 0.089919636 | 3.621701363 | 0.024828009 | downregulated | 0.000228829 |
| GHITM | 0.001 | 3.621267747 | 0.000276146 | downregulated | 0.009364413 |
| SMPDL3B | 0.266921813 | 3.575577589 | 0.074651383 | downregulated | 0.002441679 |
| ATP5O | 0.606284438 | 3.567229489 | 0.169959471 | downregulated | 0.001135711 |
| VPS35 | 0.62456221 | 3.546515043 | 0.176105896 | downregulated | 0.009032758 |
| MUC21 | 0.170084057 | 3.532283514 | 0.048151304 | downregulated | 9.09E-05 |
| LRRC15 | 0.171810731 | 3.529037585 | 0.048684869 | downregulated | 0.011706651 |
| TSPO | 0.001 | 3.511974434 | 0.00028474 | downregulated | 0.003883226 |
| EPS8 | 0.030639791 | 3.506420034 | 0.008738198 | downregulated | 0.00017033 |
| PSMD11 | 0.134661074 | 3.489204071 | 0.038593637 | downregulated | 0.009880433 |
| FRK | 0.90546078 | 3.487564224 | 0.25962555 | downregulated | 0.010313818 |
| GALE | 0.495011174 | 3.442066681 | 0.143812198 | downregulated | 0.000794937 |
| C8B | 0.001 | 3.440732191 | 0.000290636 | downregulated | 0.001282478 |
| RPL7A | 0.001 | 3.437454591 | 0.000290913 | downregulated | 0.011865591 |
| RAB18 | 0.480216381 | 3.409895536 | 0.140830232 | downregulated | 0.006006182 |
| PARK7 | 0.649428567 | 3.409838859 | 0.19045726 | downregulated | 0.010585536 |
| GSTT1 | 0.378054122 | 3.404921364 | 0.111031675 | downregulated | 0.015325841 |
| ITGAV | 0.001 | 3.403864574 | 0.000293784 | downregulated | 0.012769394 |
| PGAM4 | 1.175439416 | 3.349401898 | 0.350940094 | downregulated | 0.041823625 |
| VIM | 0.348746134 | 3.32549588 | 0.104870415 | downregulated | 0.006571208 |
| SERPIND1 | 0.340041374 | 3.26366476 | 0.104190044 | downregulated | 0.001371342 |
| TOM1 | 0.411122171 | 3.240340616 | 0.126876221 | downregulated | 0.004894244 |
| PSMD3 | 0.127340279 | 3.206353277 | 0.039714987 | downregulated | 0.000407538 |
| RPL11 | 0.715949438 | 3.165488519 | 0.226173443 | downregulated | 0.046009316 |
| GPC1 | 0.187568369 | 3.152305523 | 0.059501964 | downregulated | 0.007192615 |
| RAB3B | 0.001 | 3.118687011 | 0.000320648 | downregulated | 0.000165711 |
| GDI1 | 0.431127981 | 3.076112012 | 0.140153538 | downregulated | 0.000611446 |
| GALNT18 | 0.001 | 3.032863103 | 0.000329721 | downregulated | 0.000365223 |
| RENBP | 0.001 | 3.019698367 | 0.000331159 | downregulated | 5.62E-05 |
| RPL7 | 0.93582698 | 3.001102029 | 0.311827779 | downregulated | 0.026215461 |
| MAN2B2 | 0.247340201 | 2.998485193 | 0.082488385 | downregulated | 0.001008001 |
| PSMC6 | 0.581373994 | 2.996521664 | 0.194016282 | downregulated | 0.029452584 |
| TMEM192 | 0.001 | 2.989343283 | 0.000334522 | downregulated | 0.003361115 |
| FCGBP | 0.491202101 | 2.974892126 | 0.165115937 | downregulated | 0.009542485 |
| CHMP1A | 0.001 | 2.961964435 | 0.000337614 | downregulated | 4.66E-05 |
| SLC23A1 | 0.047957491 | 2.961181721 | 0.016195389 | downregulated | 0.002785836 |
| CCT6A | 0.816680209 | 2.958701471 | 0.276026567 | downregulated | 0.0163633 |
| FBLN1 | 0.331367024 | 2.95430372 | 0.11216417 | downregulated | 0.002032378 |
| RPSA | 1.10613059 | 2.896616825 | 0.381869835 | downregulated | 0.047478767 |
| F11 | 0.297080271 | 2.890703925 | 0.102770909 | downregulated | 0.001178085 |
| SLC2A1 | 0.073816725 | 2.886289646 | 0.025574954 | downregulated | 0.012347687 |
| REG1A | 0.001 | 2.85751908 | 0.000349954 | downregulated | 0.010353048 |
| LASP1 | 0.155314203 | 2.848082592 | 0.0545329 | downregulated | 0.00301281 |
| CAB39 | 0.497135852 | 2.837458884 | 0.175204601 | downregulated | 0.002756125 |
| HMGCS2 | 0.001 | 2.837148095 | 0.000352467 | downregulated | 0.000597334 |
| CNTFR | 0.001 | 2.827976398 | 0.00035361 | downregulated | 0.000102588 |
| PFKM | 0.780159475 | 2.818042056 | 0.276844511 | downregulated | 0.013027305 |
| DLD | 0.001 | 2.792212825 | 0.000358139 | downregulated | 0.002297371 |
| ATP6AP1 | 0.036132845 | 2.774850685 | 0.013021546 | downregulated | 0.006368969 |
| SLC5A10 | 0.097342388 | 2.774450276 | 0.035085289 | downregulated | 0.008935364 |
| EPS8L1 | 0.555616549 | 2.774305676 | 0.200272289 | downregulated | 0.008315468 |
| RPN1 | 0.642749447 | 2.761358741 | 0.232765645 | downregulated | 0.039590313 |
| PACSIN2 | 0.001 | 2.726000312 | 0.000366838 | downregulated | 0.007284543 |
| PSME2 | 0.001 | 2.717940469 | 0.000367926 | downregulated | 0.000166954 |
| ARPC1B | 0.001 | 2.716001906 | 0.000368188 | downregulated | 0.000378971 |
| C2 | 0.001 | 2.709076631 | 0.000369129 | downregulated | 8.20E-07 |
| BPIFB1 | 0.001 | 2.675281964 | 0.000373792 | downregulated | 0.018690282 |
| UQCRFS1 | 0.001 | 2.656727773 | 0.000376403 | downregulated | 0.02799501 |
| PRSS2 | 0.001 | 2.64840503 | 0.000377586 | downregulated | 0.020475275 |
| C11orf52 | 0.001 | 2.642495516 | 0.00037843 | downregulated | 0.006014239 |
| TTC38 | 0.001 | 2.631433146 | 0.000380021 | downregulated | 1.41E-05 |
| ATP6V1B2 | 0.323594102 | 2.626089352 | 0.123222807 | downregulated | 0.0054923 |
| MINPP1 | 0.158536763 | 2.603106382 | 0.060902914 | downregulated | 0.000378499 |
| CLMP | 0.001 | 2.595284795 | 0.000385314 | downregulated | 0.005100206 |
| HSD11B2 | 0.043616192 | 2.579015514 | 0.016911954 | downregulated | 0.000612706 |
| BCAP31 | 0.583766912 | 2.556180819 | 0.228374655 | downregulated | 0.019642855 |
| DBNL | 0.64395508 | 2.556158359 | 0.251922999 | downregulated | 0.047548779 |
| BAIAP2 | 0.362684227 | 2.553894629 | 0.142012213 | downregulated | 0.001554447 |
| TGM4 | 0.001 | 2.546604784 | 0.00039268 | downregulated | 0.009031386 |
| HSPB11 | 0.001 | 2.542625772 | 0.000393294 | downregulated | 0.029482197 |
| AKR1C1 | 0.481174638 | 2.525593684 | 0.190519418 | downregulated | 0.015205399 |
| RAB3D | 0.001 | 2.525102842 | 0.000396023 | downregulated | 0.000108598 |
| RNH1 | 0.673051372 | 2.515994631 | 0.267509065 | downregulated | 0.012782158 |
| GCA | 0.001 | 2.492364868 | 0.000401225 | downregulated | 0.025548461 |
| RNPEP | 0.219696596 | 2.490797656 | 0.088203309 | downregulated | 0.000891767 |
| UQCRC1 | 0.460203926 | 2.476930814 | 0.185796036 | downregulated | 0.021189322 |
| DYNC1H1 | 0.458845742 | 2.476190129 | 0.185303114 | downregulated | 0.003973192 |
| CALB1 | 0.396393077 | 2.461341444 | 0.161047578 | downregulated | 0.026227264 |
| CS | 0.321210933 | 2.443876672 | 0.131435001 | downregulated | 0.005896307 |
| TINAGL1 | 0.001 | 2.432160064 | 0.000411157 | downregulated | 0.015475548 |
| ADH6 | 0.001 | 2.42699003 | 0.000412033 | downregulated | 9.28E-05 |
| PDIA6 | 0.377490376 | 2.418120139 | 0.156109024 | downregulated | 0.018109983 |
| OSCAR | 0.001 | 2.404803683 | 0.000415834 | downregulated | 0.041580726 |
| RAB43 | 0.001 | 2.390673216 | 0.000418292 | downregulated | 1.51E-05 |
| ARHGDIB | 0.001 | 2.379809024 | 0.000420202 | downregulated | 0.007243615 |
| AOX1 | 0.016758841 | 2.372446772 | 0.007063948 | downregulated | 0.00023436 |
| CTNNA1 | 0.651998442 | 2.356810344 | 0.276644425 | downregulated | 0.036073384 |
| DDT | 0.001 | 2.355029191 | 0.000424623 | downregulated | 0.016595537 |
| RPS11 | 0.583875831 | 2.344643252 | 0.249025446 | downregulated | 0.007269328 |
| ASL | 0.346606495 | 2.339624917 | 0.14814618 | downregulated | 0.007010078 |
| COASY | 0.094973125 | 2.334111926 | 0.04068919 | downregulated | 0.000435943 |
| PDHA1 | 0.001 | 2.333204866 | 0.000428595 | downregulated | 0.012790438 |
| MSRA | 0.001 | 2.324956662 | 0.000430116 | downregulated | 0.002509327 |
| SERINC1 | 0.001 | 2.295786868 | 0.000435581 | downregulated | 0.001036134 |
| ALPL | 0.031960853 | 2.286365015 | 0.013978894 | downregulated | 0.027009959 |
| CAPN2 | 0.268155935 | 2.224583919 | 0.120542063 | downregulated | 0.000734058 |
| PDHB | 0.393886171 | 2.216353671 | 0.177718104 | downregulated | 0.034852276 |
| AFM | 0.001 | 2.212791855 | 0.000451918 | downregulated | 0.00345463 |
| LCN2 | 0.001 | 2.180216863 | 0.00045867 | downregulated | 0.007158997 |
| HIST1H2AA | 0.001 | 2.177000844 | 0.000459348 | downregulated | 0.001481173 |
| MYO1D | 0.135359258 | 2.175863046 | 0.062209457 | downregulated | 0.002984693 |
| TSPAN3 | 0.001 | 2.175077542 | 0.000459754 | downregulated | 0.002162492 |
| GLG1 | 0.483936328 | 2.174578985 | 0.222542538 | downregulated | 0.002976127 |
| OMD | 0.001 | 2.163966978 | 0.000462114 | downregulated | 0.014333231 |
| ALDH7A1 | 0.152952991 | 2.162836652 | 0.070718697 | downregulated | 4.38E-05 |
| TCN2 | 0.001 | 2.153495774 | 0.000464361 | downregulated | 0.004587145 |
| TECR | 0.384544338 | 2.130275945 | 0.180513862 | downregulated | 0.015591547 |
| HADH | 0.404934763 | 2.12869078 | 0.190227142 | downregulated | 0.039632543 |
| SCRN2 | 0.001 | 2.12579849 | 0.000470411 | downregulated | 7.69E-07 |
| EBP | 0 | 2.113582243 | 0 | downregulated | 0.012272899 |
| NME1 | 0.123638864 | 2.078193395 | 0.059493435 | downregulated | 0.000491923 |
| HNRNPC | 0.635321481 | 2.068478012 | 0.307144421 | downregulated | 0.04409517 |
| GBA | 0.001 | 2.062470044 | 0.000484856 | downregulated | 0.000463583 |
| HLA-DRB1 | 0.001 | 2.046868374 | 0.000488551 | downregulated | 0.003568455 |
| CAPZA1 | 0.450902807 | 2.043895627 | 0.220609507 | downregulated | 0.024296876 |
| PRKACB | 0.026755832 | 2.036415963 | 0.013138687 | downregulated | 0.000565874 |
| CD47 | 0.001 | 2.031574799 | 0.000492229 | downregulated | 0.014403163 |
| PYGL | 0.15490121 | 2.030474134 | 0.076288197 | downregulated | 0.001501459 |
| ANGPTL1 | 0.001 | 2.019657153 | 0.000495134 | downregulated | 0.003677275 |
| MAN1C1 | 0.342045899 | 2.019209735 | 0.169395924 | downregulated | 0.011166912 |
| ZMPSTE24 | 0.001 | 2.01477625 | 0.000496333 | downregulated | 0.000772848 |
| GLUD2 | 0.197876848 | 2.014715032 | 0.0982158 | downregulated | 0.001280088 |
| BST1 | 0.001 | 1.996721659 | 0.000500821 | downregulated | 0.009075717 |
| PSMD2 | 0.033112851 | 1.98916915 | 0.016646574 | downregulated | 0.000844571 |
| ATIC | 0.456529271 | 1.98796695 | 0.229646308 | downregulated | 0.020403062 |
| GPT | 0.044211831 | 1.971241471 | 0.02242842 | downregulated | 0.001985901 |
| HSD17B10 | 0.476333062 | 1.970207442 | 0.241767974 | downregulated | 0.01916963 |
| ARL6IP5 | 0.400949824 | 1.958177406 | 0.204756639 | downregulated | 0.042482251 |
| RAC3 | 0.001 | 1.950105403 | 0.000512793 | downregulated | 0.013563422 |
| SLC13A2 | 0.001 | 1.94696273 | 0.000513621 | downregulated | 0.01134197 |
| EPHB4 | 0.001 | 1.946289723 | 0.000513798 | downregulated | 0.028675675 |
| WASL | 0.117051847 | 1.942475595 | 0.060259108 | downregulated | 0.000830939 |
| GSTM5 | 0.001 | 1.928465359 | 0.000518547 | downregulated | 0.004366712 |
| VPS29 | 0.001 | 1.919775727 | 0.000520894 | downregulated | 0.008639207 |
| CORO1A | 0.001 | 1.912169604 | 0.000522966 | downregulated | 0.002402025 |
| AXL | 0.001 | 1.911352254 | 0.00052319 | downregulated | 0.007858281 |
| ART3 | 0.001 | 1.893927339 | 0.000528003 | downregulated | 0.030830558 |
| PRKACA | 0.057815769 | 1.872928989 | 0.030869173 | downregulated | 0.008735474 |
| LOC101060453 | 0 | 1.854373624 | 0 | downregulated | 0.008714805 |
| ATP6V0A1 | 0.010442372 | 1.839147098 | 0.005677834 | downregulated | 0.000690136 |
| PVRL1 | 0.001 | 1.837503341 | 0.000544217 | downregulated | 0.016812099 |
| APEH | 0.001 | 1.818033094 | 0.000550045 | downregulated | 0.000306317 |
| NAPRT1 | 0.260977533 | 1.804761398 | 0.144605006 | downregulated | 0.000619235 |
| ANGPTL6 | 0.289162745 | 1.799945807 | 0.160650806 | downregulated | 0.01322459 |
| COPB2 | 0.453213815 | 1.797348546 | 0.252156888 | downregulated | 0.004361973 |
| RPS23 | 0.001 | 1.794836463 | 0.000557154 | downregulated | 0.00024259 |
| DYNLL1 | 0.001 | 1.794807943 | 0.000557163 | downregulated | 0.001454072 |
| SLC4A4 | 0.001 | 1.791933938 | 0.000558056 | downregulated | 0.002155481 |
| CD82 | 0.001 | 1.783025366 | 0.000560845 | downregulated | 0.022743349 |
| SLC5A2 | 0.147436387 | 1.777043408 | 0.08296724 | downregulated | 0.000388097 |
| HK3 | 0.001 | 1.776816393 | 0.000562804 | downregulated | 0.000817175 |
| GALC | 0.046318139 | 1.746277256 | 0.026523932 | downregulated | 4.26E-05 |
| H2AFX | 0.001 | 1.735691756 | 0.000576139 | downregulated | 0.004660834 |
| C21orf33 | 0.001 | 1.733768257 | 0.000576778 | downregulated | 0.018948057 |
| GPC4 | 0.001 | 1.713368878 | 0.000583645 | downregulated | 5.01E-05 |
| KRT38 | 0.001 | 1.70689333 | 0.00058586 | downregulated | 0.023265863 |
| KRT23 | 0.001 | 1.700434808 | 0.000588085 | downregulated | 0.022009566 |
| SLC5A1 | 0.001 | 1.696498426 | 0.000589449 | downregulated | 0.00571011 |
| PTPRJ | 0.193967252 | 1.692157199 | 0.114627206 | downregulated | 0.002698968 |
| ALDH3A1 | 0.001 | 1.686561853 | 0.000592922 | downregulated | 0.040698901 |
| ACSM2B | 0.001 | 1.658922133 | 0.000602801 | downregulated | 0.00026148 |
| LOC101060798 | 0.090925885 | 1.658712853 | 0.054817134 | downregulated | 0.004061899 |
| MNDA | 0.011664627 | 1.643596638 | 0.007097013 | downregulated | 0.024111483 |
| ICOSLG | 0.001 | 1.641947526 | 0.000609033 | downregulated | 0.038261852 |
| ERMP1 | 0.001 | 1.638328219 | 0.000610378 | downregulated | 0.046188755 |
| GSTM2 | 0.191177847 | 1.601851952 | 0.119348012 | downregulated | 0.005351472 |
| ACSM2A | 0.001 | 1.583681575 | 0.00063144 | downregulated | 0.000115161 |
| EPX | 0.001 | 1.581239955 | 0.000632415 | downregulated | 0.019015848 |
| ARL8B | 0.086032653 | 1.560662966 | 0.055125709 | downregulated | 0.001431004 |
| TRIP10 | 0.001 | 1.559668337 | 0.000641162 | downregulated | 0.004143483 |
| GIPC1 | 0.138999649 | 1.558385983 | 0.089194622 | downregulated | 0.000763432 |
| CAT | 0.217480853 | 1.549832048 | 0.140325433 | downregulated | 0.016190587 |
| ATP6V1C1 | 0.28694643 | 1.546159362 | 0.185586581 | downregulated | 0.031705587 |
| ABCB4 | 0.382150563 | 1.545426291 | 0.247278414 | downregulated | 0.011853234 |
| LRRK2 | 0.001 | 1.54438127 | 0.000647509 | downregulated | 0.002113152 |
| UBE2N | 0.001 | 1.542778364 | 0.000648181 | downregulated | 0.021996225 |
| CTNNB1 | 0.505113856 | 1.523099192 | 0.331635561 | downregulated | 0.018569999 |
| PAH | 0.001 | 1.516882384 | 0.000659247 | downregulated | 0.000216496 |
| C19orf10 | 0.001 | 1.490412547 | 0.000670955 | downregulated | 0.007323031 |
| SCIN | 0.001 | 1.482425059 | 0.00067457 | downregulated | 0.000855459 |
| NPR3 | 0.016389372 | 1.48165997 | 0.011061493 | downregulated | 0.002252157 |
| ENPP3 | 0.001 | 1.479331219 | 0.000675981 | downregulated | 0.024866295 |
| DNM2 | 0.325623911 | 1.474633805 | 0.220816795 | downregulated | 0.008958605 |
| PCDH1 | 0.001 | 1.465028071 | 0.000682581 | downregulated | 0.015467993 |
| PTPRG | 0.027163899 | 1.464140559 | 0.018552794 | downregulated | 0.000399759 |
| PCDHGC3 | 0.001 | 1.463924302 | 0.000683095 | downregulated | 0.002589433 |
| CTNND1 | 0.240220931 | 1.462132151 | 0.164294952 | downregulated | 0.014370931 |
| TMED9 | 0.140669605 | 1.461954774 | 0.096220217 | downregulated | 0.00298223 |
| STEAP4 | 0.001 | 1.460293945 | 0.000684794 | downregulated | 0.011108897 |
| TREH | 0.001 | 1.454477298 | 0.000687532 | downregulated | 0.005524239 |
| MTCH2 | 0.001 | 1.452336198 | 0.000688546 | downregulated | 0.017095193 |
| ACOX3 | 0.128391166 | 1.451907032 | 0.08842933 | downregulated | 0.041824006 |
| ARHGAP1 | 0.470442448 | 1.428441261 | 0.329339722 | downregulated | 0.018590979 |
| ATP6V0D1 | 0.001 | 1.421074681 | 0.000703693 | downregulated | 0.001952569 |
| HIST2H2AB | 0.001 | 1.419897411 | 0.000704276 | downregulated | 0.007089967 |
| SLC47A1 | 0.001 | 1.405201498 | 0.000711642 | downregulated | 0.00102861 |
| CFL2 | 0.001 | 1.39873272 | 0.000714933 | downregulated | 0.000436404 |
| GFPT1 | 0.117450533 | 1.389705336 | 0.084514703 | downregulated | 0.005053153 |
| AKR1C4 | 0.053663192 | 1.38054131 | 0.038871124 | downregulated | 0.007682461 |
| ARPC5 | 0.001 | 1.379129308 | 0.000725095 | downregulated | 0.010259079 |
| ST6GAL1 | 0.344989477 | 1.376952987 | 0.250545575 | downregulated | 0.040160888 |
| H2AFV | 0.001 | 1.368732514 | 0.000730603 | downregulated | 0.010597783 |
| CORO1C | 0.018778786 | 1.367001192 | 0.013737212 | downregulated | 0.019673418 |
| RAC2 | 0.001 | 1.361928678 | 0.000734253 | downregulated | 0.001898044 |
| NAAA | 0.001 | 1.348846976 | 0.000741374 | downregulated | 0.000313476 |
| AP1G1 | 0.03416315 | 1.346915873 | 0.025363982 | downregulated | 0.012216492 |
| ITLN2 | 0.001 | 1.326518515 | 0.000753853 | downregulated | 0.028632464 |
| SLC25A1 | 0.022817813 | 1.308588968 | 0.017436959 | downregulated | 0.00704396 |
| DNASE2 | 0.001 | 1.302862812 | 0.000767541 | downregulated | 0.005066382 |
| CD84 | 0.001 | 1.294309261 | 0.000772613 | downregulated | 0.000154506 |
| TSPAN4 | 0.001 | 1.284345379 | 0.000778607 | downregulated | 1.40E-06 |
| GP5 | 0.126885601 | 1.279067438 | 0.099201651 | downregulated | 0.004040293 |
| PGM3 | 0.001 | 1.266486338 | 0.000789586 | downregulated | 0.007157637 |
| ARL15 | 0.001 | 1.257358337 | 0.000795318 | downregulated | 0.00048565 |
| SIRPA | 0.001 | 1.253319897 | 0.000797881 | downregulated | 0.027672294 |
| SPON2 | 0.001 | 1.248022416 | 0.000801268 | downregulated | 0.019158543 |
| CTSL | 0.001 | 1.242691991 | 0.000804705 | downregulated | 0.002971479 |
| RPS20 | 0.001 | 1.240636782 | 0.000806038 | downregulated | 0.001478956 |
| HIBCH | 0.086634785 | 1.220835384 | 0.070963527 | downregulated | 0.0034643 |
| DDOST | 0.231425089 | 1.215298668 | 0.190426514 | downregulated | 0.035391028 |
| PSMA5 | 0.199647042 | 1.211102779 | 0.164847315 | downregulated | 0.006434403 |
| GAMT | 0.001 | 1.200441838 | 0.000833027 | downregulated | 0.008333845 |
| FAM3C | 0.001 | 1.19819921 | 0.000834586 | downregulated | 3.57E-07 |
| CSF1 | 0.001 | 1.189538259 | 0.000840662 | downregulated | 3.36E-06 |
| SIRPB1 | 0.001 | 1.181529937 | 0.00084636 | downregulated | 0.002600947 |
| FKBP4 | 0.001 | 1.169233876 | 0.000855261 | downregulated | 0.002248469 |
| IL18BP | 0.001 | 1.165759526 | 0.00085781 | downregulated | 0.040685526 |
| OGDH | 0.214960501 | 1.158787417 | 0.18550469 | downregulated | 0.005940495 |
| C5 | 0.001 | 1.140891374 | 0.000876508 | downregulated | 0.021260776 |
| GPD1L | 0.001 | 1.13071736 | 0.000884394 | downregulated | 0.000638042 |
| SUCLA2 | 0.001 | 1.126407978 | 0.000887778 | downregulated | 0.007124418 |
| ITFG1 | 0.001 | 1.126021985 | 0.000888082 | downregulated | 0.000276746 |
| SPAG9 | 0.001 | 1.122944563 | 0.000890516 | downregulated | 3.72E-05 |
| GLS | 0.001 | 1.113273053 | 0.000898252 | downregulated | 0.001029404 |
| SULT1A1 | 0.001 | 1.1104753 | 0.000900515 | downregulated | 1.91E-05 |
| GOLM1 | 0.001 | 1.101757152 | 0.000907641 | downregulated | 0.019131319 |
| CTTN | 0.0342156 | 1.100191041 | 0.03109969 | downregulated | 0.008592516 |
| SLC22A8 | 0.001 | 1.100039271 | 0.000909058 | downregulated | 0.007400692 |
| LAMB2 | 0.001 | 1.098085649 | 0.000910676 | downregulated | 0.027701511 |
| ALDH8A1 | 0.001 | 1.090037244 | 0.0009174 | downregulated | 0.000444869 |
| MAPK3 | 0.025478417 | 1.089583387 | 0.023383632 | downregulated | 0.030285976 |
| PPT1 | 0.001 | 1.08562561 | 0.000921128 | downregulated | 0.002969551 |
| NRP1 | 0.001 | 1.084764752 | 0.000921859 | downregulated | 0.001236548 |
| IQGAP2 | 0.001 | 1.075225889 | 0.000930037 | downregulated | 0.004384952 |
| PARD6B | 0.001 | 1.066196249 | 0.000937914 | downregulated | 0.018019389 |
| CYFIP1 | 0.153489584 | 1.055853932 | 0.145370093 | downregulated | 0.003423994 |
| LOC101060751 | 0.001 | 1.054213986 | 0.000948574 | downregulated | 0.000805422 |
| VNN1 | 0.236294886 | 1.052751456 | 0.224454581 | downregulated | 0.048435812 |
| TMBIM1 | 0.001 | 1.048980105 | 0.000953307 | downregulated | 0.001217301 |
| PSMC2 | 0.049089174 | 1.047073413 | 0.046882265 | downregulated | 0.000373357 |
| HTRA1 | 0.001 | 1.036203102 | 0.000965062 | downregulated | 0.001686579 |
| H2AFZ | 0.001 | 1.035695993 | 0.000965534 | downregulated | 0.011875214 |
| HIST1H1B | 0.15418819 | 1.028144434 | 0.149967441 | downregulated | 0.020947935 |
| PPP2R1B | 0.001 | 1.023361028 | 0.000977172 | downregulated | 0.002887756 |
| FOLH1 | 0.001 | 1.021366791 | 0.00097908 | downregulated | 0.003212188 |
| CHMP6 | 0.116434963 | 1.020811342 | 0.114061197 | downregulated | 0.018780381 |
| PSMD13 | 0.292527755 | 1.018384733 | 0.287246799 | downregulated | 0.035780771 |
| GLIPR2 | 0.001 | 1.016661962 | 0.000983611 | downregulated | 0.000350213 |
| HLA-B | 0.115610996 | 1.015369968 | 0.113860956 | downregulated | 0.002744035 |
| IGFBP6 | 0.001 | 1.002647141 | 0.00099736 | downregulated | 0.005743054 |
|  |  |  |  |  |  |
